# Candidate Interaction Partners of Calpain-5 Suggest Clues to Its Involvement in Neovascular Inflammatory Vitreoretinopathy

**DOI:** 10.3390/cells15020142

**Published:** 2026-01-13

**Authors:** Jozsef Gal, Vimala Bondada, Rachel Crasta, Dorothy E. Croall, Calvin P. Vary, James W. Geddes

**Affiliations:** 1 Spinal Cord and Brain Injury Research Center (SCoBIRC), University of Kentucky, Lexington, KY 40536, USArachel.crasta@uky.edu (R.C.); jgeddes@uky.edu (J.W.G.); 2 Department of Neuroscience, University of Kentucky, Lexington, KY 40536, USA; 3 Department of Radiation Medicine, University of Kentucky, Lexington, KY 40536, USA; 4 Markey Cancer Center, University of Kentucky, Lexington, KY 40536, USA; 5 Department of Molecular and Biomedical Sciences, University of Maine, Orono, ME 04469, USA; croall@maine.edu; 6 Graduate School of Biomedical Sciences and Engineering, University of Maine, Orono, ME 04469, USA; 7 Center for Molecular Medicine, MaineHealth Institute for Research, Scarborough, ME 04074, USA; calvin.vary@mainehealth.org

**Keywords:** CAPN5, interaction, proteomics, membrane, substrate, neurodegeneration, disease, vitreoretinopathy, NIV

## Abstract

**Highlights:**

**What are the main findings?**

- Fifty-one candidate interaction partners of calpain-5/CAPN5 were identified in neuroblastoma cells.
- Many candidate CAPN5 interactors are associated with the chaperome and protein quality control complexes.

**What is the implication of the main finding?**

- The findings provide hints regarding both the physiological and pathological roles of CAPN5.

**Abstract:**

Although calpain-5/CAPN5 is widely expressed in mammals, little is known regarding its functions. Pathogenic mutations of CAPN5 are causal for a devastating autoimmune eye disease, neovascular inflammatory vitreoretinopathy (NIV). To provide insight into both the physiological and pathological roles of CAPN5, it is essential to identify candidate interaction partners and possible substrates. Human SH-SY5Y neuroblastoma cells, transfected with full-length catalytically dead (Cys81Ala) CAPN5-3×FLAG, were used for anti-FLAG co-immunoprecipitation (co-IP) and quantitative proteomics using Sequential Window Acquisition of all THeoretical mass spectra (SWATH-MS). Fifty-one proteins were enriched at least four-fold, *p* < 0.01, relative to cells transfected with an empty FLAG vector. A high proportion (24/51) of candidate CAPN5 interaction partners are associated with protein quality control, including components of the chaperonin, chaperone, and ubiquitin–proteasome systems. Additional candidate interactors include tubulins, kinases, phosphatases, G proteins, and mitochondrial proteins. CAPN5 interactions for 14 of the candidate proteins were confirmed by co-IP and immunoblotting. Of these 14 proteins, 11 exhibited in vitro calcium-induced proteolysis following co-IP with WT CAPN5-3×FLAG. Impaired calcium-induced proteolysis of co-IP proteins was observed for the pathogenic CAPN5 variants R243L and R289W. Further studies are needed to validate the association of candidate CAPN5 interactors with proteins and complexes suggested by the SWATH-MS and co-IP results, and the possible role of CAPN5 within such complexes. The possible involvement of CAPN5 in protein quality control is relevant to NIV, as defects in protein quality control have been implicated in inherited retinal disorders. Proteomic data are available via ProteomeXchange with identifier PXD068008.

## 1. Introduction

Calpains, a family of intracellular calcium-dependent cysteine proteases, modulate cellular processes and signaling via limited proteolysis of substrate proteins [1,2,3]. The mammalian genome encodes 15 calpains defined by the presence of a conserved cysteine protease core domain [2,3,4]. Classical calpains (calpains 1, 2, 3, 8, 9, 11, 12, 13, 14) are characterized by both a protease core domain and a penta-EF hand domain [3,5]. Calpain-5 (CAPN5) contains a protease core domain, but the penta-EF hand domain is replaced by a C-terminal C2 domain with the potential to bind calcium, and is categorized as a non-classical calpain [6,7]. Much less attention has been given to CAPN5 as compared to classical calpains, particularly calpains 1 and 2, and comparatively little is known regarding the interaction partners and substrates of CAPN5.

CAPN5 is ubiquitously, although differentially, expressed in various cells and tissues [6,8]. The *CAPN5* mRNA is one of the most highly expressed among calpains in the CNS [8,9], and is also highly expressed in the gastrointestinal tract, kidney, liver, testis, and trachea [6]. In the retina, *CAPN5* mRNA expression is modest relative to calpains 2 and 7 [10]. At the subcellular level, CAPN5 is primarily localized to the cytoplasm and plasma membrane, but is also detected in the nucleus, mitochondria, and endoplasmic reticulum [9,10,11,12,13]. In the retina, immunocytochemistry reveals CAPN5 localization to the inner and outer segments of photoreceptors and the outer plexiform layer, with lower levels in some retinal ganglion cells, the inner nuclear layer, and inner plexiform layer [10]. Subcellular fractionation confirms CAPN5 expression in the rod outer segment, although most remained in the “rest of retina” fraction, including soluble membrane and synaptic fractions [10]. CAPN5 membrane localization is facilitated by its C2 domain and likely stabilized by S-acylation [7,14]. Calcium-induced CAPN5 activation appears to predominantly involve the S-acylated membrane-associated enzyme [7,14,15].

Individual classical calpain isoforms have specific protein substrates which are proteolyzed at specific residues. However, mechanisms involved in substrate recognition are incompletely understood [16,17,18,19]. Unlike the caspases these substrates bear no evident consensus sequences. With the exception of CAPN15 [20], there are no known post-translational modifications such as ubiquitination that target the substrates for proteolysis. The location of specific cleavage sites appears to depend on both the primary sequence and secondary structure of the target proteins [17]. Substate preference of calpains is thought to be determined by isoform-specific gating loops in the protease core domain that regulate substrate access to the catalytic site. The CAPN5 gating loops are elongated relative to those of classical calpains, presumably resulting in the recognition of distinct substrates [21,22].

A rare but devastating eye disease, neovascular inflammatory vitreoretinopathy (NIV), is caused by mutations in CAPN5 which alter its subcellular localization and activity [11,15,22,23,24]. To better understand the physiological roles of CAPN5 and the mechanisms by which pathogenic mutations cause NIV, it is important to identify CAPN5 interaction partners and substrates.

Previously reported proteolytic substrates of CAPN5 include SLIT2 [25], caspase-4 [13], AIRE [15], and CAPN5 itself as an autoproteolytic substrate [7,14]. Using N-terminomics, an additional twenty-nine potential CAPN5 substrates (twenty-four unique proteins and members of five protein groups) were recently identified [26]. Large-scale affinity purification–mass spectrometry (AP-MS) studies using CAPN5 as bait identified STIP1 as an interactor [27,28]. CAPN5 was listed as a possible prey/target of several additional proteins in studies curated by BioGrid [29,30] (Table S1). Overall, there is little to no overlap regarding candidate CAPN5 interaction partners and substrates identified in previous studies.

The primary goal of this study was to expand our view of candidate interaction partners and putative substrates of CAPN5 as a necessary step to elucidate CAPN5 physiologic functions. A secondary goal was to obtain clues to the pathologic consequences of CAPN5 variants causal for NIV. Methods included co-immunoprecipitation (co-IP)/affinity purification (AP) from SH-SY5Y human neuroblastoma cells stably expressing catalytically inactive C81A mutant CAPN5-3×FLAG or empty vector control cells followed by quantitative proteomic analysis with sequential window acquisition of all theoretical fragment ion spectra (SWATH-MS) [31,32,33]. Fifty-one proteins were significantly enriched at least four-fold (*p* < 0.01) and largely represent novel candidate CAPN5 interaction partners. Fourteen candidate interactors, along with six additional proteins with links to retinal and neurologic disorders, were further evaluated by co-IP of endogenous or HA-tagged candidate proteins with CAPN5-3×FLAG followed by incubation with calcium and immunoblotting for evidence of proteolysis. Select pathogenic CAPN5 variants associated with NIV (R243L, R289W, G267S) were also evaluated for calcium-induced proteolysis.

## 2. Materials and Methods

### 2.1. Cell Culture and the Generation of Stable Cell Lines

The human neuroblastoma cell line SH-SY5Y (ATCC, Manassas, VA, USA, CRL-2266 [34]) was cultured in MEM (Corning, Corning, NY, USA, 10-010-CV) with 10% fetal bovine serum (Atlanta Biologicals, Flowery Branch, GA, USA, S11195H) and penicillin-streptomycin (Corning, 30-002-CI) under 5% CO_2_/95% air at 37 °C in a humidified cell culture incubator. The cells were transfected with pIRESpuro3-C81A CAPN5-3×FLAG or the pIRESpuro3-3×FLAG control using LipoJet (Signagen, Frederick, MD, USA, SL100468), and stable cell lines were generated and maintained with 3 μg/mL puromycin (Gold Biotechnology, St. Louis, MO, USA, P-600-100).

### 2.2. Plasmid Construction

#### 2.2.1. CAPN5-3×FLAG Expression Constructs

The construction of the pIRESpuro3-C81A CAPN5-3×FLAG and the WT, C81A, R243L, G267S, and R289W CAPN5-3×FLAG plasmids based on p3×FLAG-CMV-14 (Sigma, St. Louis, MO, USA, E4901) were reported previously [7,14,15]. The pIRESpuro3-3×FLAG control plasmid was made by isolating the *Sac*I-*Bam*HI fragment encoding the 3×FLAG tag from p3×FLAG-CMV-10 (Sigma, E4401), and inserting it between identical sites of pIRESpuro3 (Takara/Clontech, San Jose, CA, USA, Catalog #631619).

#### 2.2.2. HA-Tagged Protein Expression Constructs

The pCGN N-Ras WT plasmid expressing N-terminally HA-tagged NRAS [35] was obtained from Addgene (Watertown, MA, USA) (a gift from Adrienne Cox, Addgene plasmid #14723). The p3×HA-Nterm expression vector was made by replacing the *Sac*I-*Hin*dIII small fragment of p3×FLAG-CMV-10 with the annealed oligonucleotides 5′-CGTTTAGTGAACCGTCAGAATTAACCATGGCTTACCCATACGATGTTCCAGATTACGCTGGATCTTACCCATACGATGTTCCAGATTACGCTGGATCTTACCCATACGATGTTCCAGATTACGCTA-3′ and 5′-AGCTTAGCGTAATCTGGAACATCGTATGGGTAAGATCCAGCGTAATCTGGAACATCGTATGGGTAAGATCCAGCGTAATCTGGAACATCGTATGGGTAAGCCATGGTTAATTCTGACGGTTCACTAAACGAGCT-3′. The N-terminal 3×HA-tagged expression constructs for TUBB4B and G protein subunit beta 3 (GNB3) were generated by PCR amplification of the respective coding sequences from templates obtained from DNASU (Tempe, AZ, USA) [36,37,38] and inserting them between the *Eco*RI-*Xba*I sites of p3×HA-Nterm, as indicated in Table 1.

The p3×HA-Cterm expression vector was made by replacing the *Bam*HI-*Xma*I fragment encoding the 3×FLAG tag sequence of p3×FLAG-CMV-14 with annealed oligonucleotides, as reported previously [14]. The C-terminal 3×HA-tagged GNAT1, GNAT2, LYN, RDH11, and SPTLC1 expression constructs were generated by PCR amplification of the respective coding sequences from templates obtained from DNASU and inserting them between the *Eco*RI-*Xba*I sites of p3×HA-Cterm, as indicated in Table 1.

The N-terminal 3×HA-tagged GNB2 expression construct was made by Gateway recombination of the entry clone obtained from DNASU (Table 2) and the pGCS-N2 (3×HA) destination vector (a gift from Hai-Ning Du, Addgene plasmid #85719) [39].

The C-terminal 3×HA-tagged GNAI2, ATP1A1, and RHO expression constructs were made by Gateway recombination of the respective entry clones obtained from DNASU (Table 2) and the destination vector pCSF107mT-GATEWAY-3′-3HA (a gift from Todd Stukenberg, Addgene plasmid #67616). The SCRIB-3×HA construct was made by Gateway recombination of the entry clone R777-E306 Hs.SCRIB-nostop (a gift from Dominic Esposito, Addgene plasmid #70590) and pCSF107mT-GATEWAY-3′-3HA. All Gateway reactions were carried out with the LR Clonase II Enzyme Mix (Thermo Fisher Scientific, Waltham, MA, USA, 11791020).

The HA-tag sequences were identical in the 3×HA-tagging expression vectors (YPYDVPDYA), but with differences in spacing. In p3×HA-Cterm and p3×HA-Nterm, glycine–serine linkers separated the HA-tags. In pGCS-N2 (3×HA), the HA-tags have no linkers. In pCSF107mT-GATEWAY-3′-3HA, a glycine residue separates the first and second HA-tags, and a glycine–serine linker separates the second and third HA-tags.

All plasmid constructs were confirmed with sequencing.

### 2.3. Co-Immunoprecipitation

The identification of CAPN5 interaction partners with co-IP was based on the comparison of three independent biological replicates of anti-FLAG immunoprecipitates from C81A CAPN5-3×FLAG stable cell and control cell lysates using SWATH-MS analysis (see below). The steps were performed on ice, unless otherwise indicated. Confluent cultures of C81A CAPN5-3×FLAG stable SH-SY5Y cells and control cells were rinsed twice with phosphate-buffered saline (PBS) [26], and lysed on the plates with lysis buffer (50 mM Tris-HCl, pH 7.5, 150 mM NaCl, 1 mM EDTA, 1% Triton-X100) supplemented with protease inhibitors (Sigma, P8340, 1:300) and PhosSTOP phosphatase inhibitor (Sigma, 4906837001, 1 tablet/10 mL). The lysates were homogenized by passing through 23G needle. The lysates were shortly vortexed at 10 and 20 min. The lysates were cleared (centrifugation at 8200× *g*, 4 °C, 10 min). The supernatants were transferred to fresh tubes, and the total protein concentrations were measured with Protein Assay Dye Reagent (Bio-Rad, Hercules, CA, USA, 5000006). Anti-FLAG M2 magnetic beads (Sigma, M8823) were washed with lysis buffer. The lysates were adjusted to identical protein concentrations with lysis buffer, and subjected to immunoprecipitation with the anti-FLAG beads for 3 h at 4 °C. The magnetic beads were washed three times with 30 bead volumes of lysis buffer containing protease and phosphatase inhibitors as above. Proteins were eluted with two changes of 5× packed bead volumes of elution buffer (0.1 M glycine-HCl, pH 3.0) at room temperature for 5 min each. The eluates were transferred to low protein binding tubes (Thermo Fisher Scientific, Cat. #90410) pre-aliquoted with 1/10 eluate volume of 1 M Tris-HCl, pH 8.5, and 1/30 eluate volume of 5 M NaCl, and frozen on dry ice.

### 2.4. Affinity Capture

The identification of CAPN5 interaction partners with affinity capture was based on the comparison of eluates from C81A mutant CAPN5 catalytic core affinity resin and control resin (technical triplicates generated from two independent experiments). Expression of full-length CAPN5 in *E. coli* generated predominantly insoluble product extractable with 8 M urea (AJ Bolduc & DE Croall, unpublished observations). Instead, the far more soluble maltose binding protein (MBP)–CAPN5 catalytic core (CC) domain (Phe2-Leu348) with C81A mutation fusion construct (MBP-CAPN5-C81A-CC) was used as the bait in the affinity capture experiments. Overnight cultures of BL21 (DE3) *E. coli* (EMD Millipore, Burlington, MA, USA, Cat. #69450) transformed with MBP-CAPN5-C81A-CC were diluted 1:100 into fresh Luria broth (LB) in the presence of carbenicillin (100 µg/mL), and growth at 37 °C was monitored at A600. Isopropyl-beta-D-thiogalactoside (IPTG, 0.5 mM) was added to chilled cultures with A600 = 0.6 – 0.8 and grown overnight at room temperature. The cells were harvested by centrifugation, and washed with 0.15 M NaCl prior to suspension in 20 mM MOPS, pH 7.5, 0.2 M NaCl, 2 mM EGTA, 10 mM magnesium acetate, 5 mM 2-mercaptoethanol (the recommended amylose resin binding buffer), and frozen at −20 °C. The cell suspension was thawed and lysed by gentle sonication in amylose binding buffer with protease inhibitor (PMSF, 50 µg/mL). The supernatant from centrifugation (20,000× *g*, 4 °C, 20 min) was applied to an affinity column of pre-equilibrated amylose resin (New England Biolabs, Ipswich, MA, USA, E8021). The resin was washed with amylose binding buffer. Unbound proteins and wash fractions were discarded. The elution was performed with 10 mM maltose in amylose binding buffer lacking protease inhibitors. The buffer was exchanged to 20 mM MOPS, pH 8.0, 0.2 M NaCl, 5 mM imidazole, pH 8.0, 0.1 mM EGTA, and 0.1 mM 2-mercaptoethanol using Amicon centrifugal concentrators with 30 kDa molecular weight cutoff (EMD Millipore). Nickel affinity chromatography was performed with Ni-NTA agarose (Qiagen, Germantown, MD, USA, Cat. #30230) using standard methods with elution by addition of 0.25 M imidazole to the buffer. The elution buffer was exchanged as above with 30 mM MOPS, pH 7.0, 0.15 M KCl, 0.5 mM dithiothreitol, and 5 mM magnesium acetate (MKD buffer + Mg) for the final isolated bait protein. SDS-PAGE of the protein recovered after both affinity chromatography steps showed a single major protein at 88 kDa, near the predicted size of 87 kDa. Protein concentration was assessed by using A280 with the theoretical extinction coefficient of the protein. Aliquots of the isolated bait protein were stored at 4 °C for use within two weeks, or immediately flash frozen in liquid nitrogen, and stored at −80 °C. Samples were thawed and maintained at 4 °C 24 h prior to experiments.

Lysates of SH-SY5Y cells for affinity capture were prepared as follows. Low-passage-number parental SH-SY5Y cells were cultured to confluence. The cells were collected with trypsinization, washed with 1× PBS, and frozen at −80 °C until use. Lysates were prepared by thawing cells in hypotonic buffer (20 mM MOPS, pH 7.5, 4 mM EGTA, 4 mM EDTA, 0.1 mM 2-mercaptoethanol) with protease inhibitor (PMSF, 1 mM). After initial homogenization (Ten Broeck glass homogenizer, Corning, 7727-2), Triton X-100 was added to 1% (*v*/*v*), and the lysates were re-homogenized. The lysate was cleared with centrifugation (20,000× *g*, 4 °C, 15 min), and adjusted to 30 mM MOPS, pH 7.5, 0.15 M KCl, 2 mM magnesium acetate (MKM buffer), 0.05 mM 2-mercaptoethanol, and 0.1% Triton X-100.

The affinity capture protocol was based on reagent manufacturer’s recommendations (Chromotek now Proteintech, Rosemont, IL, USA) with all steps performed at 4 °C, unless noted otherwise. MBP-Trap agarose beads (mbta, 70 µL suspension, Proteintech) were blocked with 1 mg/mL BSA in MKM buffer with end-over-end rotation for 2 h. The beads were washed with 30 mM MOPS, pH 7.5, and 0.15 M KCl using centrifuge columns (Thermo Fisher Scientific, 89868) prior to end-over-end rotation with SH-SY5Y lysate for 2 h. Unbound proteins were recovered to serve as the pre-cleared lysate for affinity capture by immobilized MBP-CAPN5-C81A-CC (see below). The control resin was washed twice with MKM buffer supplemented with 0.1% Triton-X100, followed by two washes with MKM buffer (50 bead volumes each wash) prior to elution of bound proteins with three changes of 0.2 M glycine, pH 2.5 at room temperature. The pooled glycine-eluted samples were neutralized with 1 M Tris base.

MBP-Trap agarose beads were used to immobilize MBP-CAPN5-C81A-CC. The bait protein at 0.4 µM (17 µg protein in 0.5 mL) in the presence of 1 mg/mL BSA in MKM buffer was added to pre-washed MBP-Trap beads (70 µL suspension) and incubated with end-over-end rotation for 2 h. The excess MBP-CAPN5-C81A-CC bait and BSA were removed using centrifuge columns (Thermo Fisher Scientific, 89868), and the bait-immobilized resin was washed with MKM buffer prior to incubation with the pre-cleared lysate for 2 h with end-over-end rotation. MBP-CAPN5 bait was ~0.2 µM during incubation with prey proteins. The MBP-Trap resin was washed with MKM-0.1% Triton-X100 and MKM (50 bead volumes each) prior to elution with three changes of 0.2 M glycine, pH 2.5. Pooled elution samples were neutralized with 1 M Tris base.

### 2.5. SWATH-MS Analysis

Our SWATH workflow was described in detail previously [40]. Briefly, the proteins in the eluates were precipitated with ethanol, then reduced with dithiothreitol, followed by capping of the thiol groups with iodoacetamide. The proteins were digested with trypsin. The tryptic peptides were isolated on C18 reverse phase spin columns. Analytical peptide separation was performed on a high resolution C18 reverse phase chromatography column on an Ultimate RSLC system 3000 (Thermo Fisher Scientific/Dionex, Sunnyvale, CA, USA) nanoscale liquid chromatograph and infused onto a 5600 TripleTOF mass spectrometer (SCIEX, Framingham, MA, USA). For proteomics informatics, an isotope-free unbiased scanning workflow, SWATH-MS [31,32,41], was used. Human-specific ion libraries comprising 1089 proteins were constructed using the ProteinPilot program package (Version 5.0.2, SCIEX). To identify peptides, multiple fragment ion chromatograms were retrieved from the spectral library for the peptides of interest. The spectra were compared to extracted fragment ion traces for the respective isolation window to identify transitions that best corresponded to the target peptides. Based on these data, lists of proteins and their corresponding relative levels with appropriate statistics were generated by our software workflow that included the SCIEX programs ProteinPilot (data-dependent peptide ion libraries), PeakView, version 2.2 (linking data-independent SWATH MS/MS analysis and ion library data, quantification of peaks, determination of the false discovery rate, quality control analytics, via the SWATH microapp, version 2.0.1), and MarkerView, version 1.3 (principle component analysis [42], *p*-values, *t*-test comparisons). The mass spectrometry data have been deposited with ProteomeXchange via PRIDE [43] with the dataset identifier PXD068008.

### 2.6. Confirmatory co-IPs and the In Vitro CAPN5 Assay

The co-IPs and the in vitro CAPN5 assay were described previously [15]. Briefly, SH-SY5Y cells were transfected with expression constructs for 3×FLAG-tagged WT, C81A, R243L, G267S, or R289W CAPN5, or the 3×FLAG vector control, with or without expression constructs for 3×HA-tagged interaction partners. Two days later, lysates were prepared in 1× RIPA buffer (Sigma, Cat. #20-188) supplemented with AEBSF and phosphatase inhibitors. The lysates were subjected to immunoprecipitation with anti-FLAG beads. The proteins were eluted among native conditions with 3×FLAG peptide. The eluates were transferred to low protein binding tubes in two equal aliquots. In one aliquot, CAPN5 was activated by the addition of 3 mM calcium chloride. After a 2 h incubation at room temperature, the reactions were stopped by the addition of SDS-PAGE loading buffer and heating at 60 °C.

The confirmatory co-IPs and in vitro CAPN5 assays were performed as single experiments with extensive controls. The expression of the 3×FLAG-tagged CAPN5 baits and the 3×HA-tagged prey proteins were controlled by their respective empty vectors. The expression of the endogenous prey proteins was confirmed with immunoblotting of the lysates with specific antibodies. The intrinsic affinity of the prey proteins to the anti-FLAG resin was determined by anti-FLAG immunoprecipitation of lysates from cells that were transfected with the insert-free 3×FLAG vector control. The electrophoretic mobilities of the full-length proteins corresponded to the expectations. The calcium activation in the in vitro CAPN5 assays were confirmed by CAPN5 autolysis shown by the loss of full-length WT, but not C81A CAPN5, and also by the appearance of low molecular weight C-terminal autolytic CAPN5-3×FLAG fragments [26]. These autolytic fragments are apparent on the high-brightness anti-FLAG immunoblots of the calcium-treated eluates, submitted as Supplementary Data. The C81A mutant served as a positive control for the CAPN5 interaction, but as negative control for the CAPN5 proteolytic activity. Anti-actin immunoblotting served as control for lysate loading.

### 2.7. Denaturing Protein Gel Electrophoresis and Immunoblotting

Denaturing gel electrophoresis and immunoblotting were performed as reported before [26]. Briefly, equal volumes of the FLAG immunoprecipitates and equal protein amounts of the lysates were resolved on 3–8% or 4–12% gradient protein gels. The resolved proteins were transferred to nitrocellulose membranes. The primary antibodies are summarized in Table 3. The secondary antibodies were Alexa Fluor 680 goat anti-mouse IgG (Thermo Fisher Scientific, A-21058, 0.2 µg/mL) and IRDye 800CW goat anti-rabbit IgG (Li-Cor, Lincoln, NE, USA, 926-32211, 0.1 µg/mL). The immunoblots were scanned on an Odyssey CLx Imaging System (Li-Cor) using Image Studio, Version 5.2. Adjustments to brightness and contrast were applied equally to entire images. Blot lanes were not spliced from different images. Composite images were assembled with Adobe Illustrator 2023 (Version 27.4.1) and Adobe Photoshop 2023 (Version 24.3.0).

### 2.8. Gene Ontology and Databases

Gene Ontology (GO) analysis [44,45] was performed using GO::TermFinder v0.86 [46] accessed at https://go.princeton.edu/, on 1 August 2025. For the GO analysis background, five SH-SY5Y proteomes [47,48,49,50,51] were compiled into a single compendium of 7338 proteins. Forty-eight proteins identified in the present study but not included in the five previous proteomic studies were added for a total of 7386 proteins (Table S2). GO-annotated protein clusters were considered significantly enriched with a corrected *p* value of less than 0.01 and greater than four-fold enrichment, relative to the SH-SY5Y proteome background.

Data regarding lipid modifications and subcellular localization were gathered from the UniProt database (https://uniprot.org/, release 22 April 2025 [52]) and the SwissPalm database (https://swisspalm.org/, release 5, 9 April 2025) [53,54]). The Human Protein Atlas database (https://proteinatlas.org/) [55] was also utilized to gather human cell- and tissue-specific expression information (version: 23.0, updated: 9 June 2023). The Biological General Repository for Interaction Datasets (BioGRID) v4.4.247 [29,30] provided data on previous studies of protein interactions of CAPN5 and candidate interaction partners.

## 3. Results

Following anti-FLAG immunoprecipitation of lysates of SH-SY5Y cells stably transfected with C81A CAPN5-3×FLAG, SWATH-MS analysis identified 1088 co-immunoprecipitated proteins, of which 51 exhibited greater than four-fold enrichment relative to 3×FLAG vector immunoprecipitated controls and were statistically significant at *p* < 0.01. CAPN5 and the 51 putative interaction partners were manually sorted based on function (Table 4). The full results are in Table S3 and are also available via ProteomeXchange with identifier PXD068008.

Many of the candidate CAPN5 interaction partners (24/51) are associated with protein quality control systems including translation, proper protein folding, refolding of misfolded proteins, and protein degradation. This includes chaperonins, chaperones and heat shock proteins, and members of the ubiquitin–proteasome system and endoplasmic reticulum-associated degradation (ERAD). Also identified were several tubulin subunits and other cytoskeletal and structural proteins, as well as kinases, phosphates, G protein signaling proteins, proteins associated with RNA processing, and mitochondrial metabolic proteins.

For clarity and consistency, we utilize the gene name throughout with the abbreviated protein name added when substantially different from the gene name (i.e., STUB1/CHIP). The 51 putative CAPN5 interaction partners were submitted to the Gene Ontology GO: Term Finder (https://go.princeton.edu/) to evaluate annotations of GO Molecular Functions, Biological Processes, and Cellular Components [44,56]. Table 5 illustrates the three most significant clusters with a minimum of ten proteins within each GO category. The full list is provided in Table S4, which includes the annotated gene list for each GO term.

Consistent with the manual sorting in Table 4, the most highly significant GO clusters for each category were related to protein folding/stabilization and chaperones (Table 5). Examination of the proteins associated within each cluster (Table S4) revealed a predominance of chaperone/heat shock proteins and subunits of the Chaperonin Containing Tailless complex polypeptide 1 (TCP1) (CCT) complex, also known as the TCP1 Ring Complex (TRiC) [57,58]. Tubulin subunits were also within many clusters.

Notably, all eight members of the CCT/TRiC complex were identified as candidate CAPN5 interactors. The only CCT/TRiC subunit not identified was CCT6B/TCPW, whose expression is restricted to the testis [59]. The eight subunits of the CCT/TRiC complex form two stacked rings with a folding chamber cavity in the center to assist protein folding in an ATP-driven manner [60]. Approximately 10% of newly translated proteins may interact with the CCT/TRiC complex, including several structural and regulatory proteins with tubulin and actin being the most abundant substrates [58,61,62,63]. Several α- and β-tubulin subunits were identified as potential CAPN5 interaction partners. Cytoplasmic actin subunits ACTG1 and ACTB were identified by AP-MS-SWATH but were not enriched relative to control (Table S3).

Protein folding by CCT/TRiC is facilitated by chaperones and co-chaperones, with the collective protein folding machinery referred to as the “chaperome” [64,65,66]. In addition to CCT/TRiC subunits, human chaperome components identified as potential CAPN5 interaction partners included the HSP90 co-chaperone CDC37, the BAG family molecular chaperone regulator 2/BAG2, Heat shock protein HSP 90-alpha/HSP90AA1, Heat shock protein HSP 90-beta/HSP90AB1, Heat shock 70 kDa protein 8/HSPA8, Chaperonin 60/HSPD1, Heat shock 70 kDa protein 1B/HSPA1B, stress-induced-phosphoprotein 1/STIP1, the DnaJ homolog subfamily A members DNAJA1, DNAJA2, and DNAJA3, the DnaJ homolog subfamily C member DNAJC7, and the E3 ubiquitin–protein ligase STUB1/CHIP.

Given that full-length CAPN5 is a multi-domain protein and that it fails to fold properly in bacterial expression systems [7,19], it may require chaperones and the chaperonin system to achieve its stable conformation. However, more than half of the proteins identified by co-IP were also affinity captured using a purified, bacterially expressed maltose binding protein (MBP) fusion to the CAPN5-Cys81Ala-catalytic core domain (Phe2-Leu348) as bait from lysates of untransfected SH-SY5Y cells. SWATH analysis of replicate experiments identified 33 of the 51 targets captured by co-IP to be enriched >two-fold with *p* < 0.05, providing supporting evidence for the interaction of those proteins with the catalytic domain of CAPN5 (Table S5, data available through ProteomeXchange, identifier PXD071887). Thirteen of those proteins including two tubulins (TUBB, TUBA1B) and four TRiC subunits (TCP1, CCT2, CCT3, CCT4) were enriched >four-fold, *p* < 0.005 (Table S5). This suggests that the observed interactions with chaperones and chaperonins are not simply due to their roles in folding CAPN5.

**Figure 1:**
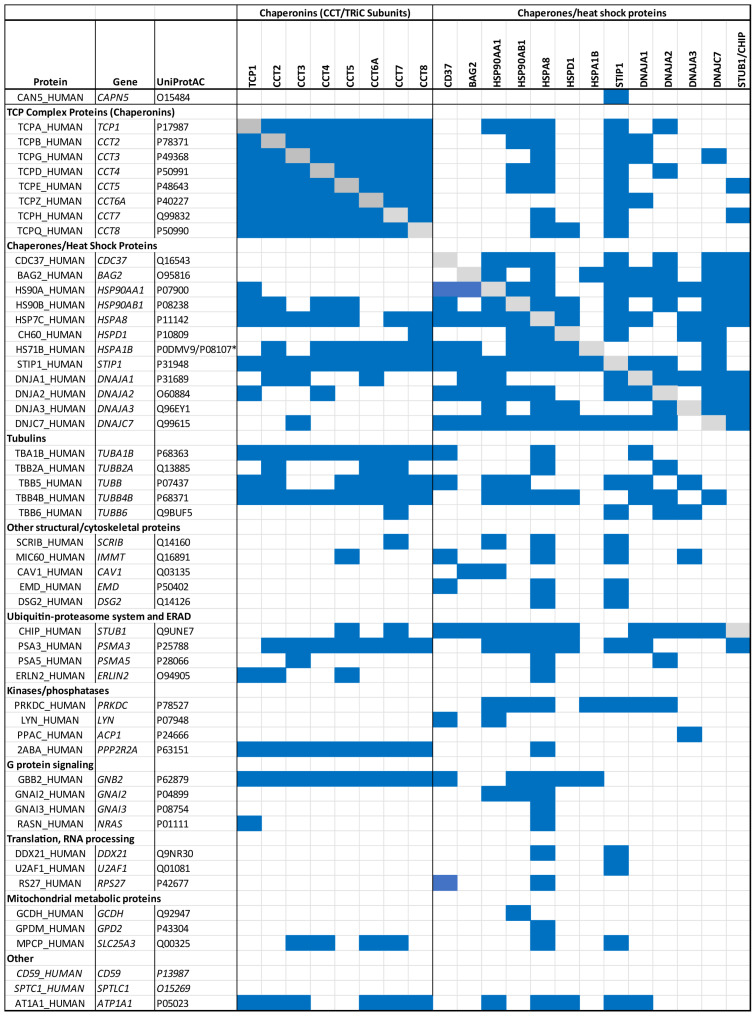
Putative CAPN5 interaction partners and the interactomes of chaperome components. Identification of a putative CAPN5 interaction partner in the interactome of a chaperome component (top row) is indicated in blue. Self-interactions are excluded (denoted in gray). The two putative CAPN5 interaction partners not identified in chaperome interactomes are italicized. *: UniProtAC P08107 is an obsolete term which referred to both *HSPA1A* and *HSPA1B*. This UniProt entry has been demerged and replaced by *P0DMV8* (*HSPA1A*) and *P0DMV9* (*HSPA1B*). However, several of the chaperome component interactomes in BioGRID include the P08107 entry.

To further explore the relationship between chaperome components and the 51 candidate CAPN5 interactors, we examined the interactome of the identified chaperome components using the BioGRID database, v4.4 [29] (Figure 1). The eight CCT/TRiC subunits (chaperonins) and twelve chaperones/heat shock proteins interact extensively with each other and with many of the remaining members of the CAPN5 interactome. Only two proteins, the membrane attack complex inhibition factor CD59 and serine palmitoyltransferase 1/SPTLC1, were not found in the interactomes of the chaperome members identified as CAPN5 interaction partners in this study (Figure 1).

Identification of eight CCT/TRiC subunits as candidate CAPN5 interactors was striking. Several of the CCT/TRiC subunits are S-acylated, as is CAPN5, and S-acylation has been implicated in protein complex assembly [14,53]. We therefore sought to determine if other putative CAPN5 interaction partners might also be S-acylated. To investigate this idea, the 51 putative CAPN5 interaction partners were submitted to the SwissPalm database (Release 5, https://swisspalm.org/) which incorporates results of large scale palmitoyl-proteome studies [53,54]. Each of the 51 candidate CAPN5 interaction partners were identified in one or more palmitoyl-proteomes from human cells and tissues (Table S6). Forty-eight of the 51 candidate CAPN5 interactors were identified as being S-acylated using both metabolic labeling and chemical modification techniques, which enhances confidence [53]. In comparison, 54% of the proteins in the SH-SY5Y proteome were identified as potentially S-acylated in the SwissPalm database. In total, 13 of the 51 (25%) candidate CAPN5 interactors were validated as being S-acylated in targeted studies, as compared to six percent of human proteins. The interactors validated as being S-acylated are five CCT/TRiC subunits [53], HSP90AA1 [67], G protein α subunits GNAI2 and GNAI3 [68], GTPase NRAS [69], the Scribble homolog SCRIB [70], Tyrosine–protein kinase LYN [71,72], Desmoglein-2/DSG2 [73], and Caveolin-1/CAV1 [74]. We previously validated CAPN5 S-acylation by acyl-PEG exchange [14], although it has not yet been incorporated into the SwissPalm database. Together these results suggest the possibility that S-acylation of CAPN5 and of many candidate interactors may contribute to their association in protein complexes.

**Figure 2:**
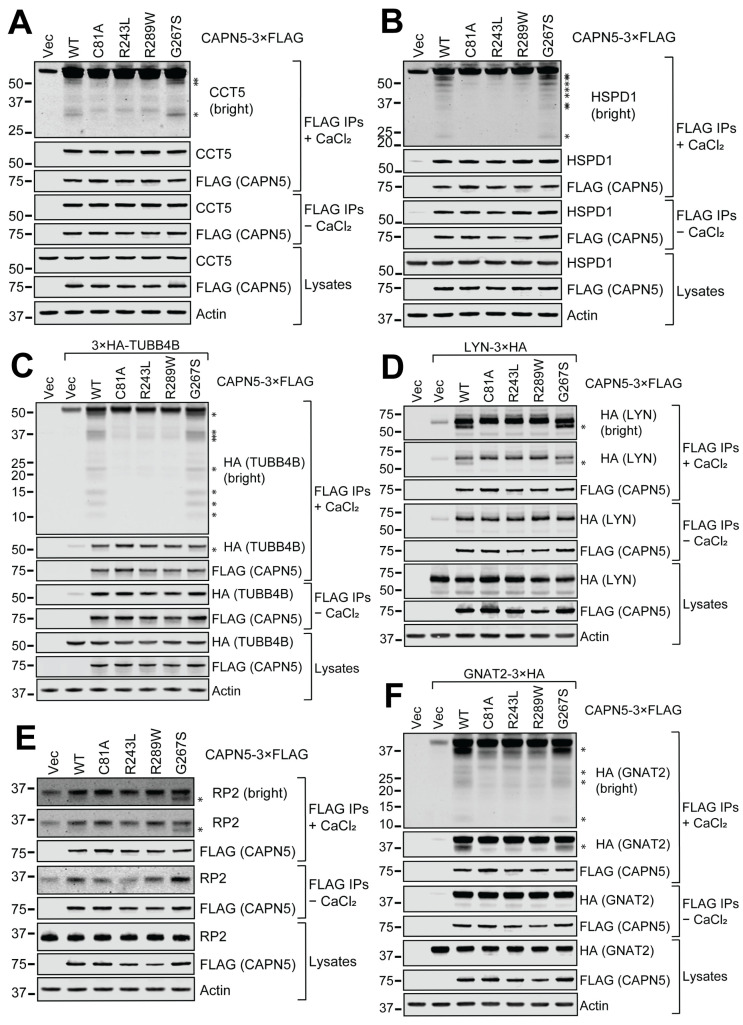
In vitro CAPN5 assays of selected CAPN5 interaction partners identified in this study: (**A**) CCT5; (**B**) HSPD1; (**C**) 3×HA-TUBB4B; (**D**) LYN-3×HA; (**E**) RP2; (**F**) GNAT2-3×HA. SH-SY5Y cells were transfected with the indicated expression constructs or their respective vector controls. The cellular lysates were subjected to anti-FLAG immunoprecipitation, and the bound proteins were eluted among native conditions with 3×FLAG peptide. Aliquots of the eluates were incubated with or without activating CAPN5 by the addition of CaCl_2_ followed by denaturing protein gel electrophoresis and immunoblotting with the indicated antibodies. Prominent proteolytic fragments are denoted by asterisks. The bars indicate molecular weight marker bands (kDa).

It is important to confirm potential interactors identified by proteomics by other techniques such as immunoblotting of co-IP samples. Fourteen candidate CAPN5 interactors were selected for further evaluation including an in vitro CAPN5-mediated proteolysis assay. The interactors were selected as representatives of functional categories in Table 4 as well as their linkage to ocular and neurologic disorders with potential relevance to NIV (Table 6). Proteolytic substrates of CAPN5 are expected to be CAPN5 interactors, but not all interaction partners are substrates. For these studies, cells expressing active (WT) CAPN5-3×FLAG were used to allow testing of susceptibility to proteolysis in the presence of added calcium. The results of the confirmatory co-IPs and the CAPN5 assays are summarized in Figure 2 and Figures S1–S17, and Table 7.

Co-IP with WT CAPN5-3×FLAG bait followed by immunoblotting confirmed the interaction of CAPN5 with nine unique endogenous proteins: CCT5, HSPD1/CH60, the DNA-dependent protein kinase catalytic subunit PRKDC, the sodium/potassium-transporting ATPase subunit alpha-1/ATP1A1, endoplasmic reticulum lipid raft-associated protein 2/ERLIN2, SCRIB, STIP1, STUB1/CHIP, and SPTLC1 (Figure 2A,B and Figure S1–S7). Co-IP of endogenous β-tubulin was demonstrated using an antibody that does not discriminate between individual β-tubulin isoforms (Figure S8). We were not able to obtain a commercially available antibody specific to the tubulin beta-4B chain. However, the TUBB4B interaction was confirmed with an HA-tagged expression construct and anti-HA immunoblotting (Figure 2C). Co-IP and immunoblotting confirmed the interaction between both endogenous (Figure S2) and ATP1A1-3×HA (Figure S9). Co-IP enrichment was relatively low for endogenous SCRIB (Figure S4), but much higher for overexpressed SCRIB-3×HA (Figure S10). The polyclonal antibody against SPTLC1 appeared to also cross-react with a higher molecular weight protein, therefore HA-tagged SPTLC1 was evaluated (Figure S7). The co-IP of endogenous LYN, GNAI2, G protein subunit beta-2/GNB2, and NRAS were not detected with immunoblotting, potentially due to their low levels. However, the co-precipitation of their overexpressed HA-tagged derivatives (LYN-3×HA, GNAI2-3×HA, 3×HA-GNB2, and HA-NRAS) with WT CAPN5-3×FLAG were confirmed (Figure 2D and Figures S11–S13).

The in vitro proteolysis of the co-precipitating WT CAPN5-3×FLAG interaction partners was evaluated by supplementing the eluates with CaCl_2_, as described previously [15,26]. CAPN5 activation was confirmed by monitoring CAPN5 autoproteolysis as described previously [14,15]. Calcium-induced proteolysis was not observed for any candidate protein following co-IP with catalytically dead C81A CAPN5-3×FLAG and calcium incubation, indicating that the observed calcium-induced proteolysis was the result of CAPN5 activation and was not due to other calpains or proteases.

Endogenous proteins CCT5, HSPD1, and PRKDC exhibited proteolysis following immunoprecipitation with WT CAPN5-3×FLAG and calcium incubation, as evidenced by detection of lower molecular weight breakdown products and the loss of intact protein (Table 7, Figure 2A,B and Figure S1). Calcium-induced proteolysis of β-tubulin was observed using both a non-isoform specific antibody against endogenous β-tubulin and exogenously expressed 3×HA-TUBB4B (Figure 2C and and Figure S8, respectively). Calcium incubation resulted in a loss of full-length ATP1A1, ERLIN2, and SCRIB relative to controls; however, breakdown products were not detected (Figures S2–S4). Calcium-induced fragmentation was observed for SPTLC1-3×HA, ATP1A1-3×HA, SCRIB-3×HA, and LYN-3×HA (Figure 2D, Figures S7, S9 and S10). WT CAPN5-induced fragmentation of GNAI2-3×HA was detected, although complicated by the presence of bands that were also present with non-proteolytic C81A CAPN5 control (Figure S11).

In contrast to the calcium-induced proteolysis of proteins outlined above, neither the loss of full-length protein nor the appearance of proteolytic fragments was detected following calcium incubation of co-immunoprecipitated STIP1, STUB1, 3×HA-GNB2, and HA-NRAS (Figures S5, S6, S12 and S13).

Also evaluated were three pathogenic variants of CAPN5 reported to be causal for NIV: R243L, G267S, and R289W [11,15,22,78]. Following calcium incubation, dramatically reduced proteolytic activity towards the co-precipitating CAPN5 interaction partners was observed for the R243L and R289W CAPN5 variants. In contrast, calcium-induced proteolytic activity of the G267S variant was typically similar to that of WT CAPN5. However, for some proteins such as CCT5 and β-tubulin, the intensity of the proteolytic products was greater for G267S as compared to WT CAPN5 (Figure 2A and Figure S8), consistent with the enhanced activity of this variant observed previously [15,22].

The above results demonstrated in vitro CAPN5 proteolysis of heterotrimeric G protein α-subunit GNAI2, but not β-subunit GNB2. In view of the importance of G protein signaling in the retina [79,80], we sought to determine if selective CAPN5 proteolysis of α vs. β G protein subunits was also applicable to the retina-specific Transducin heterotrimeric G protein [79,81]. The 3×HA-tagged α-subunits GNAT1 and GNAT2 were cleaved by CAPN5, whereas the 3×HA-tagged GNB3 β-subunit was not (Table 8, Figure 2F, Figures S14 and S15). GNAT1 and GNAT2 are retina-specific proteins. GNB3 is retina-enriched and was not detected in SH-SY5Y cells in the present study.

We also investigated Rhodopsin/RHO, Protein XRP2/RP2, and Retinol dehydrogenase 11/RDH11. Rhodopsin, a G protein-coupled photoreceptor consisting of the opsin protein RHO and 11-cis-retinal, represents the initial event in phototransduction in rod cells in the retina [82]. Pathogenic rhodopsin variants cause autosomal dominant retinitis pigmentosa 4 [83,84,85]. Early stages of NIV can resemble retinitis pigmentosa [11,86]. Another form of retinitis pigmentosa, x-linked retinitis pigmentosa 2, is caused by mutations in the *RP2* gene encoding the XRP2 protein, a GTPase activating protein [87,88]. Retinol dehydrogenase 11/RDH11 converts 11-cis-retinol back to 11-cis-retinal in the retinal pigmented epithelium [89]. RDH11 mutations underlie another form of retinal dystrophy [90]. RHO is a retina-specific protein. RP2 and RDH11 were identified in SH-SY5Y cells by our AP-MS-SWATH, although outside of the selection criteria (RP2 3.6× enrichment, *p* = 0.002; RDH11 4.4× enrichment, *p* = 0.11). RDH11-3×HA and RP2 were proteolyzed by CAPN5 following calcium incubation, but RHO-3×HA was not, although they all co-precipitated with CAPN5-3×FLAG (Table 8, Figure 2E, Figures S16 and S17).

In summary of the twenty proteins evaluated by in vitro analysis, fourteen were susceptible to CAPN5 mediated proteolysis and six were resistant. For examples in which both the endogenous and the 3×HA-tagged proteins were evaluated, the results obtained were consistent.

## 4. Discussion

SH-SY5Y human neuroblastoma cells are widely used for research on neuronal cell biology and are well characterized [34,91,92]. They were chosen for the present study for the reasons outlined below. CAPN5 is ubiquitously, although differentially, expressed in a variety of cell types. The SH-SY5Y cell line expresses CAPN5 mRNA at a relatively high level (https://proteinatlas.org/). The initial report of endogenous CAPN5 activation was in SH-SY5Y cells [8]. Our previous studies have utilized SH-SY5Y cells to evaluate CAPN5 localization, calcium-induced activation, role of the C2 domain in membrane localization and activation, and the contribution of S-acylation to stabilizing the membrane localization [7,14,15,26]. Based on the previous characterization of CAPN5 in SH-SY5Y cells, this cell line is an appropriate model system for the primary goal of this study: to identify candidate CAPN5 interaction partners and possible substrates.

The secondary goal of our study was to provide clues to the pathologic consequences of CAPN5 variants causal for NIV. Although ocular problems are associated with NIV, the R289W variant is also associated with hearing loss and developmental delay, indicating that the consequences of pathogenic CAPN5 variants are not restricted to the retina and ocular systems [24,25,93]. The initial clinical symptoms of NIV (Stage I) typically resemble autoimmune uveitis and a reduction in the b-wave on electroretinograms [86,94,95]. The a-wave is not affected early in the course of the disease. The disrupted b-wave is thought to originate from bipolar and/or Müller glial cells, whereas the intact a-wave arises from photoreceptor outer segments [96]. This suggests that the pathogenesis of NIV may not only affect the photoreceptor outer segment. NIV progression to Stage II involves pigmentary photoreceptor degeneration, similar to retinitis pigmentosa. Subsequent stages involve retinal neovascularization, retinal detachment, and vitreous hemorrhage [94,95,97]. With NIV pathogenesis involving multiple components (autoimmunity, inflammation, neovascularization, and neurodegeneration), the use of a cell line with properties of both neurons and non-neural cells is appropriate for investigations into the consequences of pathogenic CAPN5 mutations.

Fifty-one candidate CAPN5 interaction partners were identified in SH-SY5Y cells via affinity purification utilizing C81A CAPN5-3×FLAG as bait, followed by SWATH-MS analysis. AP-MS is widely used to identify protein–protein interactions as well as protein complexes [98,99,100,101]. Use of the SWATH workflow provides quantitative rigor and statistical significance. The identified interactors may include direct interactions between bait and prey as well as indirect interactions with other proteins in a complex [100,102]. Distinguishing between direct and indirect CAPN5 interaction partners requires further studies. The proteolytic substrates of CAPN5 are expected to be CAPN5 interactors, but not all interaction partners are substrates. The identified interaction partners that were found to be in vitro proteolytic substrates of CAPN5 are more likely to be direct interaction partners of CAPN5.

Almost half of the candidate CAPN5 interactors identified in this study are members of protein complexes associated with protein quality control systems linked to proper protein folding, refolding of misfolded proteins, and protein degradation. This includes eight subunits of the CCT/TRiC complex/chaperonin system, chaperones including members of both the HSP90 and HSP70 heat shock protein families, and components of the ubiquitin–proteasome system [103,104,105,106,107]. Protein quality control systems interact extensively to maintain functional protein homeostasis (proteostasis) [65,108]. In a recent N-terminomics/Terminal amine isotopic labeling of substrates (TAILS) study, the HSP90 Heat shock proteins HSP90AA1 and/or HSP90AB1 were identified as potential CAPN5 substrates [26]. This further suggests that CAPN5 is not simply a client protein of the protein quality control system.

Previous AP-MS studies using epitope-tagged CAPN5 as bait identified stress-induced phosphoprotein 1 (STIP1) as an interacting protein [27,28], and STIP1 was among the candidate CAPN5 interactors in the present study as well. The CAPN5-STIP1 interaction was confirmed by CAPN5-3×FLAG IP, followed by anti-STIP1 immunoblotting (Figure S5). The STIP1 interactome includes 31 of the 51 candidate CAPN5 interactors. STIP1, also referred to as HSC70/HSP90-organizing protein (Hop), is a co-chaperone for HSP70 and HSP90 [109], consistent with the identification of HSP90 (HSP90AA1, HSP90AB1) and HSP70 (HSPA8 and HSPA1B) proteins as candidate interactors. Each of these chaperones/heat shock proteins have large interactomes, based on BioGrid curation. When the STIP1 interactome is combined with that of the four heat shock proteins, 49 of the 51 candidate CAPN5 interactors are represented (Figure 1).

The interactomes of CCT/TRiC subunits, tubulin isoforms, and GNB2 also included several candidate CAPN5 interactors. Tubulin subunits are preferred substrates for the CCT/TRiC complex [63]. CAPN6, which similarly to CAPN5 has a C2 domain but is non-proteolytic, binds and stabilizes microtubules via its C-terminal domains [6,110,111]. TUBB4B/TUBB8 and TBB6 were also recently identified as potential CAPN5 substrates via N-terminomics [26]. G protein complexes require CCT/TRiC for assembly [112,113]. Thus, CAPN5 interaction with STIP1, CCT/TRiC subunits, tubulins, or G proteins, could result in many of the candidate interactions. In a study of mice lacking the fragile X protein (FMR1), weighted gene co-expression network analysis identified a protein module network containing CAPN5, ten tubulins, and three CCT/TRiC subunits (CCT2, CCT3, CCT4), further suggesting a functional link between CAPN5, the TRiC complex, and tubulins [114].

CAPN5 interacted with several proteins of the ubiquitin–proteasome degradation system. Typically, calpains produce limited proteolysis of target substrate proteins, with the cleavage products often targeted for degradation via the ubiquitin–proteasome system [115,116]. This is best characterized in the sarcomere, where calcium-dependent activation of classical calpains 1, 2, and 3 represents an initial step in the turnover of many sarcomere proteins [117]. The ubiquitin–proteasome system requires proteins to be in a monomeric state prior to degradation [118]. In the sarcomere, calpains remove proteins from the myofibrils and promote the depolymerization of filaments, enabling E3 ubiquitin ligases to mark the proteins for degradation via the proteasome [119,120,121,122,123,124,125,126]. Support for a similar role for CAPN5 includes the identification of Proteasome subunit alpha type-3/PSMA3, Proteasome subunit alpha type-5/PSMA5, E3 ubiquitin–protein ligase STUB1/CHIP, and the endoplasmic reticulum-associated degradation-linked ERLIN2 as candidate CAPN5 interactors (Table 4). Whereas ERLIN2 was found to be an in vitro substrate of CAPN5 (Figure S3), we could only confirm STUB1/CHIP as a CAPN5 interaction partner (Figure S6). The E3 ubiquitin ligase HUWE1 was also identified, but was outside of the 4-fold enrichment selection criteria (2.8-fold enrichment, *p* < 0.01). Previous large-scale AP-MS studies have also identified E3 ubiquitin ligases and ubiquitin ligase complex proteins as candidate CAPN5 interactors (PARK2, CUL3, KCTD7, FBXO7, TRIM67) [67,127,128,129].

The mechanisms underlying the association of CAPN5 with the candidate interactors are not known. One possible candidate is S-acylation which influences protein localization, conformation, ER export, stability, protein–protein interaction, signal transduction, and the organization of proteins into complexes [53,130,131,132,133] S-acylation is a reversible post-translational modification which involves attachment of a long-chain fatty acid, often palmitate (palmitoylation), to select cysteine residues of a protein [134]. Each of the candidate CAPN5 interaction partners was identified in one or more high-throughput palmitoyl proteome screens, curated by SwissPalm [53,54]. However, metabolic labeling with palmitate may miss stably S-acylated proteins resulting in an underestimate, while false-positives are a concern for acyl-biotin exchange and acyl-resin assisted capture technologies which identify thioester bonds, including those not related to S-acylation [53,132]. Thus, not all proteins identified in palmitoyl-proteomes are S-acylated and some S-acylated proteins may not be identified as such. Only a small fraction of proteins identified as potentially palmitoylated have been validated in targeted follow-up studies. Among the 51 CAPN5 interaction partners identified in our AP-MS-SWATH study, the proteins confirmed to be S-acylated in follow-up studies were five CCT/TRiC subunits [53], HSP90AA1 [67], GNAI2 and GNAI3 [68], NRAS [69], SCRIB [70], LYN [71,72], DSG2 [73], and CAV1 [74]. Although the results suggest that CAPN5 interactors may be enriched in S-acylated proteins, validation of S-acylation is necessary for the remaining candidate interactors.

The results support the possibility that CAPN5 associates with multimeric protein complexes including those involved in protein quality control. There are, however, alternative possible interpretations as discussed below, and further study is needed to validate the interactions suggested by the AP-MS-SWATH and co-IP studies. Given the abundance of chaperome proteins in immortalized human cells, there is a concern that prey protein abundance may contribute to the presence of candidates found [65,135]. However, many of the most abundant proteins in SH-SY5Y cells, such as actins and vimentin, were not significantly enriched and several of the candidate CAPN5 interactors are of low abundance in undifferentiated SH-SY5Y cells [51].

The AP-MS-SWATH studies were conducted under conditions designed to prevent substrate proteolysis by using a catalytically dead bait (C81A mutant CAPN5-3×FLAG) and minimizing proteolysis by other calpains/calcium-dependent proteases by chelating endogenous calcium with EDTA. Calcium is required for activation of classical calpains, with calcium binding resulting in small but significant discrete conformational changes causing the active site in the calcium-bound protease core to resemble that of non-calcium-dependent cysteine proteases such as papain and cathepsins [136]. However, the impact of calcium on substrate binding by non-classical calpains is less well understood. The influence of calcium on the CAPN5 interactome will require further investigation.

Although the calcium-dependent proteolytic role of calpains is the most widely recognized, calpains may also have non-proteolytic functions. Skeletal-muscle-specific calpain-3 interacts with, but does not proteolyze, several components of the sarcoplasmic reticulum [137]. The interactions of CAPN5 with, but lack of proteolysis of, GNB2, GNB3, NRAS, STIP1, STUB1/CHIP, and OPSD/RHO also suggest non-proteolytic functions. The G protein G_β_ subunits are also resistant to proteolysis by classical calpains [138,139]. Of particular interest is that S-acylation contributes to the membrane tethering of G_α_ subunits which are vulnerable to calpains, while G_β_ subunits are not directly attached to membranes and are resistant to CAPN5 proteolysis [68,112,140,141,142]. However, the vulnerability of S-acylated peripheral membrane proteins to CAPN5 proteolysis is not universal as S-acylation also facilitates NRAS interactions with the plasma membrane and NRAS was resistant to calcium-induced CAPN5 proteolysis [69,143,144].

NIV is a rare autoimmune retinal disorder inherited in an autosomal dominant fashion [11,86,145]. CAPN5 variants reported as causal for NIV include R243L, L244P, K250N, G267S, R289W, and G376S [11,24,78,146]. Pathogenic CAPN5 variants are reported to result in both enhanced and impaired CAPN5 activity as well as altered subcellular localization [15,22,23,24,146]. We chose to evaluate three pathogenic variants: R243L is the most common, R289W is the most severe, and the pathogenic role of G267S is less certain [11,15,93]. Our results demonstrate impaired calcium-induced proteolytic activity of the R243L and R289W CAPN5 variants against several possible substrate proteins, with the G267S variant exhibiting activity comparable to, or slightly greater than that of, WT CAPN5. These results are consistent with effects of the same pathogenic variants on CAPN5 autoproteolysis, calcium-induced CAPN5 proteolysis of AIRE, and with hyperactivity of G267S but contrast with hyperactivity reported for R243L and R289W CAPN5 variants [15,23,24]. Whether the G267S variant is necessarily causal for NIV has been questioned as it was identified in a single individual with NIV but is also found in multiple individuals without NIV [15,78]. Our current findings confirm hypoactivity of the R243L and R289W pathogenic CAPN5 variants which have been identified in multiple individuals with NIV [11,24,93,94,95]. However, our current understanding of the effect of the NIV mutations on the CAPN5 proteolytic activity has been derived from in vitro experiments that may not faithfully reproduce the in vivo conditions. Further studies are needed to determine the effect of the NIV mutations on the identified CAPN5 interaction partners in vivo.

The mechanisms by which pathogenic CAPN5 variants cause NIV are not yet known. One possibility is impaired protein quality control, defects in which have been proposed to play a major role in inherited retinal dystrophy disorders [147,148]. CAPN5 is enriched in the photoreceptor outer segment, a modified cilium consisting of a microtubular axoneme and tightly packed membranous discs and membrane proteins [10,149,150]. The outer segment is continuously renewed with distal discs shed and new discs added. Many candidate CAPN5 interactors are among proteins in the outer segment proteome and include CCT subunits, chaperones, tubulin subunits, G protein subunits, the MICOS complex subunit MIC60/IMMT, and ATP1A1 [151,152,153]. G proteins play critical roles in photoreceptor signal transduction. The CCT/TRiC complex is essential for rod outer segment biogenesis [149,154,155]. CAPN6 deficiency results in impaired ciliogenesis, potentially the result of decreased tubulin stability [156], and CAPN5 deficiency may have similar effects. LYN is involved in the regulation of both innate and adaptive immunity, and its dysfunction is associated with autoimmune conditions including autoimmune eye disease similar to the autoimmune characteristics of NIV [157,158,159]. CAV1 plays a role in retinal inflammatory modulation [160]. *RP2* mutations are associated with a form of retinitis pigmentosa which resembles early stages of NIV. Altered CAPN5 activity and localization could impact both proteolytic and non-proteolytic functions of the enzyme. Whether the underlying cause of NIV is the result of the impact of pathogenic CAPN5 mutations on a single interaction partner/substrate or multiple proteins, analogous to the multi-hit hypothesis of carcinogenesis [161,162,163], and the resultant effect on protein quality control remains to be determined.

## 5. Summary and Conclusions

The goals of this study were to (1) identify candidate interaction partners and putative substrates of CAPN5; and (2) to obtain clues regarding the pathologic consequences of CAPN5 variants causal for NIV. We identified 51 candidate CAPN5 interaction partners in the SH-SY5Y human neuroblastoma cells. Most were novel, although STIP1 was proposed as an interaction partner in previous affinity capture studies and TUBB4B, TUBB6, and HSP90AA1/HSP90AB1 were identified as potential substrates in our N-terminomics/TAILS study [26,27,28]. The 51 CAPN5 interactors represent diverse functions. Almost half were associated with protein quality control, including chaperonins, chaperones, and proteins associated with the ubiquitin–proteasome system and endoplasmic reticulum-associated degradation. The functions of additional candidate CAPN5 interactors include cytoskeleton organization, post-translational protein modification, signaling, RNA processing and translation, metabolism, and immune functions. Of 14 interactors selected for further analysis, all co-immunoprecipitated with CAPN5 and 11 underwent proteolysis following incubation of CAPN5 and the co-immunoprecipitated proteins with excess calcium. This suggests that most of the candidate interactors are also putative CAPN5 substrates, while the lack of proteolysis of some candidate interactors is consistent with non-proteolytic functions of CAPN5. We emphasize that the candidate CAPN5 interaction partners identified in this study are indeed candidates and further investigation is necessary to validate their interactions with CAPN5.

Previous studies indicated both hypo- and hyperactivity of pathogenic CAPN5 variants implicated in NIV [15,22,23]. Our current findings support hypoactivity of the R243L and R289W variants, with mild hyperactivity of G267S CAPN5. Many of the candidate interaction partners are linked to ocular and neurologic disorders with potential relevance to NIV. For example, several candidate CAPN5 interactors are associated with protein quality control, defects in which have been implicated in inherited retinal disorders [147,148]. Of interest is that CAPN1 deficiency is linked to altered protein quality control [164]. NIV is an autoimmune disease [11,86]. Dysfunction of LYN kinase, a candidate interactor, is strongly linked to autoimmune disorders [157,158,165,166]. Pathogenic variants of TUBB4B are linked to cone–rod dystrophy, retinitis pigmentosa, and sensorineural hearing loss, resembling aspects of NIV [11,86,167,168,169]. CAPN5 is localized to the photoreceptor outer segment and many candidate CAPN5 interactors are among proteins in the outer segment proteome including CCT/TRiC subunits, chaperones, tubulin subunits, and G protein subunits [10,151,152,153]. G proteins play critical roles in photoreceptor signal transduction. The CCT/TRiC complex is essential for rod outer segment biogenesis [149,154,155]. Thus, there are plausible, although speculative links to NIV for several candidate CAPN5 interactors. Whether the underlying cause of NIV is the result of the impact of pathogenic CAPN5 mutations on a single interaction partner/substrate or multiple proteins, analogous to the multi-hit hypothesis of carcinogenesis [161,162,163], remains to be determined.

## Figures and Tables

**Table 1:** The templates and amplification primers used to generate 3×HA-tagged expression constructs by PCR. All templates were obtained from DNASU. The restriction endonuclease cut sites used for the cloning (*Eco*RI, *Xba*I) are in bold/italic font.

Gene	Protein (Human)	Template	Vector	Amplification Primers
*TUBB4B*	TBB4B	HsCD00718897	p3×HA-Nterm	5′-CGTC***GAATTC***CAGGGAAATCGTGCACTTGCA-3′
				5′-CGTC***TCTAGA***CTAGGCCACCTCCTCCTCA-3′
*GNB3*	GBB3	HsCD00044032	p3×HA-Nterm	5′-CGTC***GAATTC***CGGGGAGATGGAGCAACT-3′
				5′-CGTC***TCTAGA***TCAGTTCCAGATTTTGAGGAAG-3′
*GNAT1*	GNAT1	HsCD00642120	p3×HA-Cterm	5′-CGTC***GAATTC***ACCATGGGGGCTGGG-3′
				5′-CGTC***TCTAGA***GAAGAGGCCACAGTCTTTG-3′
*GNAT2*	GNAT2	HsCD00731264	p3×HA-Cterm	5′-CGTC***GAATTC***ACCATGGGAAGTGGAGCCAGT-3′
				5′-CGTC***TCTAGA***GAAGAGGCCGCAGTCC-3′
*LYN*	LYN	HsCD00398517	p3×HA-Cterm	5′-CGTC***GAATTC***ACCATGGGATGTATAAAATCAAAAGGGAAA-3′
				5′-CGTC***TCTAGA***AGGCTGCTGCTGGTATTG-3′
*RDH11*	RDH11	HsCD00287713	p3×HA-Cterm	5′-CGTC***GAATTC***ACCATGGTTGAGCTCATGTTCCC-3′
				5′-CGTC***TCTAGA***GTCTATTGGGAGGCCCAGC-3′
*SPTLC1*	SPTC1	HsCD00622915	p3×HA-Cterm	5′-CGTC***GAATTC***ACCATGGCGACCGCCAC-3′
				5′-CGTC***TCTAGA***CACAATTGGTCCATACTGACA-3′

**Table 2:** The entry clones and destination vectors used to generate 3×HA-tagged expression constructs with Gateway cloning. The destination vectors and the SCRIB entry clone were obtained from Addgene. The other entry clones were from DNASU.

Gene	Entry Clone	Destination Vector
*GNB2*	HsCD00001017	pGCS-N2 (3×HA)
*GNAI2*	HsCD00041647	pCSF107mT-GATEWAY-3′-3HA
*ATP1A1*	HsCD00076086	pCSF107mT-GATEWAY-3′-3HA
*RHO*	HsCD00082470	pCSF107mT-GATEWAY-3′-3HA
*SCRIB*	70590	pCSF107mT-GATEWAY-3′-3HA

**Table 3:** Immunoblotting antibodies. The mouse antibodies used were all monoclonal. Most rabbit antibodies were polyclonal, except for the monoclonal anti-STUB1/CHIP and anti-STIP1 antibodies. The epitope/antigen/immunogen information was compiled from supplier data. Aa, amino acid residue.

Protein	Company	Cat. Number	Species	Epitope/Antigen/Immunogen	Concentration
Actin (Beta)	Proteintech	66009-1-Ig	Mouse	Unspecified fusion protein	0.5 µg/mL
ATP1A1	Abcam, Waltham, MA, USA	ab7671	Mouse	Full-length protein	1.0 µg/mL
Beta-tubulin	Abcam	ab6046	Rabbit poly	Proprietary fragment of human TUBB5	0.25 µg/mL
CAPN5	Genetex, Irvine, CA, USA	GTX103264	Rabbit poly	Center region of human CAPN5	1.39 µg/mL
CCT5/TCPE	Proteintech	11603-1-AP	Rabbit poly	242–541 aa of human CCT5/TCPE	0.9 µg/mL
ERLIN2	Cell Signaling, Danvers, MA, USA	2959S	Rabbit poly	Residues surrounding Gln315 of mouse ERLIN2	0.018 µg/mL
FLAG	Sigma	F3165	Mouse	Peptide DYKDDDDK	2.0 µg/mL
HA tag	Proteintech	51064-2-AP	Rabbit poly	“Peptide”	1.6 µg/mL
HSPD1/CH60	Proteintech	15282-1-AP	Rabbit poly	225–573 aa of human HSPD1/CH60	1.3 µg/mL
PRKDC	Fortis/Bethyl, Montgomery, TX, USA	A300-517A	Rabbit poly	Between aa 2050 and 2100 (center region)	0.2 µg/mL
SCRIB	Cell Signaling	4475S	Rabbit poly	Residues surrounding Gly1237 of human SCRIB	0.026 µg/mL
SPTLC1	Proteintech	15376-1-AP	Rabbit poly	1–142 aa (N-terminus) of human SPTLC1	1.0 µg/mL
STIP1 (HOP)	Cell Signaling	5670S	Rabbit mAb	Residues surrounding Pro391 of human STIP1	0.027 µg/mL
STUB1/CHIP	Cell Signaling	2080S	Rabbit mAb	Residues around Leu36 of human STUB1/CHIP	0.04 µg/mL
RP2/XRP2	Proteintech	14151-1-AP	Rabbit poly	1–350 aa (full-length) human XRP2	0.7 µg/mL

**Table 4:** Proteins enriched 4-fold or greater, *p* < 0.01, following co-IP and SWATH-MS proteomic analysis. The proteins were manually sorted into clusters based on function**.** The index values are a measure of the abundance among the identified proteins with “1” being most abundant.

Protein (Human)	Gene	UniProtAC	Protein Name	*p*-Value	Fold Change	Index
CAN5	*CAPN5*	O15484	Calpain-5	7.40 × 10^−8^	1026.5	1
**Chaperonins**						
TCPA	*TCP1*	P17987	T-complex protein 1 subunit alpha	1.13 × 10^−5^	14.2	49
TCPB	*CCT2*	P78371	T-complex protein 1 subunit beta	4.13 × 10^−9^	17.1	43
TCPD	*CCT4*	P50991	T-complex protein 1 subunit delta	1.32 × 10^−6^	12.9	68
TCPE	*CCT5* *	P48643	T-complex protein 1 subunit epsilon	3.27 × 10^−6^	7.7	39
TCPG	*CCT3*	P49368	T-complex protein 1 subunit gamma	6.42 × 10^−8^	14.1	24
TCPH	*CCT7*	Q99832	T-complex protein 1 subunit eta	5.00 × 10^−7^	14.0	55
TCPQ	*CCT8*	P50990	T-complex protein 1 subunit theta	3.31 × 10^−6^	12.0	23
TCPZ	*CCT6A*	P40227	T-complex protein 1 subunit zeta	4.17 × 10^−7^	17.7	54
**Chaperones/Heat Shock Proteins**						
CDC37	*CDC37*	Q16543	HSP90 co-chaperone Cdc37	2.78 × 10^−5^	32.6	151
BAG2	*BAG2*	O95816	BAG family molecular chaperone regulator 2	2.08 × 10^−6^	29.7	214
HS90A	*HSP90AA1*	P07900	Heat shock protein HSP90-alpha	2.14 × 10^−5^	13.9	6
HS90B	*HSP90AB1*	P08238	Heat shock protein HSP90-beta	2.40 × 10^−4^	13.6	64
HSP7C	*HSPA8*	P11142	Heat shock cognate 71 kDa protein	9.66 × 10^−5^	9.1	19
CH60	*HSPD1* *	P10809	60 kDa heat shock protein, mitochondrial	2.90 × 10^−9^	5.5	9
HS71B	*HSPA1B*	P0DMV9	Heat shock 70 kDa protein 1B	2.90 × 10^−4^	6.5	12
STIP1	*STIP1* *	P31948	Stress-induced-phosphoprotein 1	3.30 × 10^−4^	6.0	37
DNJC7	*DNAJC7*	Q99615	DnaJ homolog subfamily C member 7	7.47 × 10^−7^	5.1	744
DNJA1	*DNAJA1*	P31689	DnaJ homolog subfamily A member 1	3.61 × 10^−6^	7.2	497
DNJA2	*DNAJA2*	O60884	DnaJ homolog subfamily A member 2	6.84 × 10^−7^	7.6	466
DNJA3	*DNAJA3*	Q96EY1	DnaJ homolog subfamily A member 3, mitochondrial	8.03 × 10^−7^	4.9	626
**Tubulins**						
TBA1B	*TUBA1B*	P68363	Tubulin alpha-1B chain	1.10 × 10^−4^	12.2	40
TBB2A	*TUBB2A*	Q13885	Tubulin beta-2A chain	5.00 × 10^−10^	4.8	397
TBB5	*TUBB*	P07437	Tubulin beta chain	1.81 × 10^−7^	10.0	18
TBB4B	*TUBB4B* *	P68371	Tubulin beta-4B chain	2.17 × 10^−5^	8.6	505
TBB6	*TUBB6*	Q9BUF5	Tubulin beta-6 chain	4.18 × 10^−8^	6.38	701
**Other structural/cytoskeletal proteins**						
SCRIB	*SCRIB* *	Q14160	Protein scribble homolog	1.99 × 10^−3^	6.4	275
MIC60	*IMMT*	Q16891	MICOS complex subunit MIC60	2.14 × 10^−7^	4.6	136
CAV1	*CAV1*	Q03135	Caveolin-1	1.78 × 10^−5^	7.9	859
EMD	*EMD*	P50402	Emerin	3.00 × 10^−4^	5.7	799
DSG2	*DSG2*	Q14126	Desmoglein-2	1.49 × 10^−15^	5.3	395
**Ubiquitin–proteasome system and endoplasmic reticulum-associated degradation (ERAD)**						
CHIP	*STUB1* *	Q9UNE7	E3 ubiquitin-protein ligase CHIP	2.10 × 10^−4^	6.6	646
PSA3	*PSMA3*	P25788	Proteasome subunit alpha type-3	1.26 × 10^−5^	4.1	454
PSA5	*PSMA5*	P28066	Proteasome subunit alpha type-5	4.84 × 10^−8^	4.7	617
ERLN2	*ERLIN2* *	O94905	Erlin-2	9.99 × 10^−7^	7.5	318
**Kinases/phosphatases**						
PRKDC	*PRKDC* *	P78527	DNA-dependent protein kinase catalytic subunit	1.40 × 10^−3^	10.6	3
LYN	*LYN* *	P07948	Tyrosine-protein kinase Lyn	1.10 × 10^−6^	6.5	536
PPAC	*ACP1*	P24666	Low molecular weight phosphotyrosine protein phosphatase	3.49 × 10^−3^	5.3	622
2ABA	*PPP2R2A*	P63151	Serine/threonine-protein phosphatase 2A 55 kDa regulatory subunit B alpha isoform	7.31 × 10^−7^	4.4	730
**G protein signaling**						
GBB2	*GNB2* *	P62879	Guanine nucleotide-binding protein G(I)/G(S)/G(T) subunit beta-2	5.22 × 10^−3^	7.0	723
GNAI2	*GNAI2* *	P04899	Guanine nucleotide-binding protein G(i) subunit alpha-2	3.19 × 10^−13^	5.6	303
GNAI3	*GNAI3*	P08754	Guanine nucleotide-binding protein G(i) subunit alpha	2.46 × 10^−9^	4.4	869
RASN	*NRAS* *	P01111	GTPase NRas	4.13 × 10^−10^	4.8	878
**Translation, RNA processing**						
DDX21	*DDX21*	Q9NR30	Nucleolar RNA helicase 2	2.50 × 10^−4^	5.3	403
U2AF1	*U2AF1*	Q01081	Splicing factor U2AF 35 kDa subunit	6.01 × 10^−7^	5.3	865
RS27	*RPS27*	P42677	Small ribosomal subunit protein eS27	8.23 × 10^−6^	5.5	754
**Mitochondrial metabolic proteins**						
GCDH	*GCDH*	Q92947	Glutaryl-CoA dehydrogenase, mitochondrial	3.23 × 10^−3^	14.5	694
GPDM	*GPD2*	P43304	Glycerol-3-phosphate dehydrogenase, mitochondrial	6.44 × 10^−3^	13.3	147
MPCP	*SLC25A3*	Q00325	Phosphate carrier protein, mitochondrial	5.30 × 10^−4^	9.5	366
**Other**						
CD59	*CD59*	P13987	CD59 glycoprotein	3.20 × 10^−4^	5.9	445
SPTC1	*SPTLC1* *	O15269	Serine palmitoyltransferase 1	3.30 × 10^−5^	4.7	467
AT1A1	*ATP1A1* *	P05023	Sodium/potassium-transporting ATPase subunit alpha-1	4.61 × 10^−10^	5.5	28

* Confirmed with co-IP and immunoblotting and evaluated as possible substrates, see below.

**Table 5:** Gene Ontology analysis. Annotations and associations are from https://geneontology.org/. Number refers to the cluster frequency, the number of candidate CAPN5 interactors or SH-SY5Y proteome components identified with each GO term. Percent was calculated with number as the numerator and the total candidate CAPN5 interactors (51) or SH-SY5Y proteome components (7338) as the denominator. Fold enrichment was calculated as the percent for CAPN5 interactors divided by that for the SH-SY5Y proteome for each GO term.

GO ID	Gene Ontology Term	Corrected *p*-Value	CAPN5 Interactors		SH-SY5Y Proteome		Fold Enrichment
			Number	Percent	Number	Percent	
**Molecular Function**							
GO:0051082	unfolded protein binding	2.68 × 10^−20^	18	35%	92	1%	28
GO:0140662	ATP-dependent protein folding chaperone	1.18 × 10^−18^	13	25%	33	0%	57
GO:0044183	protein folding chaperone	1.49 × 10^−14^	13	25%	62	1%	30
**Biologic Process**							
GO:0006457	protein folding	1.13 × 10^−15^	19	37%	175	2%	16
GO:0050821	protein stabilization	2.04 × 10^−12^	16	31%	154	2%	15
GO:0061077	chaperone-mediated protein folding	2.37 × 10^−12^	12	24%	59	1%	29
**Cellular Component**							
GO:0101031	protein folding chaperone complex	5.03 × 10^−20^	14	27%	36	0%	56
GO:0005874	microtubule	4.76 × 10^−11^	15	29%	267	4%	8
GO:0099513	polymeric cytoskeletal fiber	6.52 × 10^−11^	16	31%	399	5%	6

**Table 6:** Linkage of select candidate CAPN5 interaction partners to ocular and neurologic disorders. Data on disease association is from the Retinal Information Network database (RetNet, https://retnet.org/, as of 1 August 2025), UniProt [75], the OMIM database [76], and PubMed [77].

Protein (Human)	Gene	UniProtAC	Disease Association	OMIM	PMIDs
AT1A1	*ATP1A1*	P05023	wide spectrum: axonal neuropathies, spastic paraplegia, hypomagnesemia	618036	35110381, 36738336
CH60	*HSPD1*	P10809	spastic paraplegia 13, autosomal dominant (SPG13)	605280	11898127
CHIP	*STUB1*	Q9UNE7	spinocerebellar ataxia, multisystemic neurodegeneration	615768	24312598, 28193273
ERLN2	*ERLIN2*	O94905	spastic paraplegia 18, autosomal recessive (SPG18)	611225	21330303, 38427163
GBB2	*GNB2*	P62879	neurodevelopmental disorder with hypotonia and dysmorphic facies (NEDHYDF)	619503	31698099, 33971351, 34183358
GNAI2	*GNAI2*	P04899	pediatric encephalopathy, immune dysfunction	139360	39298586
LYN	*LYN*	P07948	autoimmune disease, autoinflammatory disease, systemic, with vasculitis (SAIDV)	165120	27571405, 36122175
PRKDC	*PRKDC*	P78527	immunodeficiency 26 with or without neurologic abnormalities (IMD26)	615966	19075392, 23722905
RASN	*NRAS*	P01111	juvenile chronic myelogenous leukemia, autoimmune lymphoproliferative syndrome	607785	17332249, 17517660
SCRIB	*SCRIB*	Q14160	neural tube defects	182940	22095531
SPTC1	*SPTLC1*	O15269	hereditary sensory and autonomic neuropathies, childhood onset ALS	162400	11242114, 32470188, 34059824
STIP1	*STIP1*	P31948	neuroprotective for Parkinson’s disease	605063	35626686, 36121476
TBB4B	*TUBB4B*	P68371	Leber congenital amaurosis, neurodegenerative disease of photoreceptor cells	617879	29198720, 39876836
TCPE	*CCT5*	P48643	sensory and demyelinating neuropathies, defective autophagy	256840	16399879, 27929117, 33076433

**Table 7:** In vitro analysis of calcium-induced proteolysis.

Gene	Protein (Human)	UniProtAC	Co-Precipitation		Calcium-Induced Proteolysis		Figure Numbers
			Endogenous	HA-Tagged	Endogenous	HA-Tagged	
*CCT5*	TCPE	P48643	Yes	ND	Yes	ND	2A
*HSPD1*	CH60	P10809	Yes	ND	Yes	ND	2B
*PRKDC*	PRKDC	P78527	Yes	ND	Yes	ND	S1
*ATP1A1*	AT1A1	P05023	Yes	Yes	Yes	Yes	S2 and S9
*ERLIN2*	ERLN2	O94905	Yes	ND	Yes	ND	S3
*SCRIB*	SCRIB	Q14160	Yes	Yes	Yes	Yes	S4 and S10
*STIP1*	STIP1	P31948	Yes	ND	No	ND	S5
*STUB1*	CHIP	Q9UNE7	Yes	ND	No	ND	S6
*SPTLC1*	SPTC1	O15269	Yes	Yes	Yes	Yes	S7
NA	β-Tubulin	NA	Yes	NA	Yes	NA	S8
*TUBB4B*	TBB4B	P68371	ND	Yes	ND	Yes	2C
*LYN*	LYN	P07948	Not detected	Yes	ND	Yes	2D
*GNAI2*	GNAI2	P04899	Not detected	Yes	ND	Yes	S11
*GNB2*	GBB2	P62879	Not detected	Yes	ND	No	S12
*NRAS*	RASN	P01111	Not detected	Yes	ND	No	S13

NA, not applicable; ND, not determined.

**Table 8:** In vitro analysis of calcium-induced proteolysis of retinal proteins.

Gene	Protein	UniProtAC	Co-Precipitation		Calcium-Induced Proteolysis		Figure Numbers
	(Human)		Endogenous	HA-Tagged	Endogenous	HA-Tagged	
*GNAT1*	GNAT1	P11488	ND	Yes	ND	Yes	S14
*GNAT2*	GNAT2	P19087	ND	Yes	ND	Yes	2F
*GNB3*	GBB3	P16520	ND	Yes	ND	No	S15
*RP2*	XRP2	O75695	Yes	ND	Yes	ND	2E
*RHO*	OPSD	P08100	ND	Yes	ND	No	S16
*RDH11*	RDH11	Q8TC12	ND	Yes	ND	Yes	S17

ND, not determined.

## Data Availability

Data is contained within the article or Supplementary Materials. Further data will be made available on request from the authors.

## Supplementary Materials

The following supporting information can be downloaded at: https://www.mdpi.com/article/10.3390/cells15020142/s1, Table S1: Previously reported interaction partners of CAPN5; Table S2: The proteome of SH-SY5Y cells; Table S3: AP-MS-SWATH analysis of the proteins that co-precipitated with C81A mutant CAPN5-3×FLAG; Table S4: GO analysis of CAPN5 interaction partners identified with 4-fold or greater enrichment, *p* < 0.01; Table S5: Overlap of 33 proteins enriched 2-fold or greater, *p* < 0.05, following affinity capture with MBP-CAPN5-C81A-CC and SWATH-MS proteomic analysis with 51 proteins identified with co-IP and SWATH-MS; Table S6: SwissPalm analysis of CAPN5 interaction partners identified with 4-fold or greater enrichment, *p* < 0.01; Figures S1–S17: In vitro CAPN5 assays of selected CAPN5 interaction partners identified in this study (Table 7 and Table 8). Supplementary figure legends are provided as a separate file. CAPN5_SWATH_uncropped_images.zip: The uncropped immunoblotting images used to prepare Figure 2 and Figures S1–S17 as a compressed folder.

## Abbreviations

The following abbreviations are used in this manuscript. Abbreviations for protein and gene names are indicated in the relevant tables.

3×FLAG tag	Peptide with the sequence Asp-Tyr-Lys-Asp-His-Asp-Gly-Asp-Tyr-Lys-Asp-His-Asp-Ile-Asp-Tyr-Lys-Asp-Asp-Asp-Asp-Lys
Aa	Amino acid residue
AEBSF	4-(2-Aminoethyl)-benzenesulfonylfluoride hydrochloride
AP-MS	Affinity purification-mass spectrometry
CNS	Central nervous system
EDTA	Ethylenediaminetetraacetic acid
EGTA	Ethylene glycol-bis(2-aminoethylether)-N,N,N′,N′-tetraacetic acid
ERAD	Endoplasmic reticulum-associated degradation
HA tag	Peptide with the sequence Tyr-Pro-Tyr-Asp-Val-Pro-Asp-Tyr-Ala
IP	Immunoprecipitation
LC/MS	Liquid chromatography and tandem mass spectrometry
MBP	Maltose binding protein
MBP-CAPN5-C81A-CC	MBP-tagged C81A mutant calpain-5 catalytic domain (Phe2-Leu348)
MOPS	3-(N-Morpholino)propanesulfonic acid
NIV	Neovascular inflammatory vitreoretinopathy
PBS	Phosphate-buffered saline
PMSF	Phenylmethanesulfonyl fluoride
SDS-PAGE	Denaturing protein gel electrophoresis
SWATH	Sequential Window Acquisition of All Theoretical Mass Spectra
TAILS	Terminal amine isotopic labeling of substrates
Tris	2-Amino-2-(hydroxymethyl)-1,3-propanediol
WT	Wild type
