# Supplementary material for: Candidate Interaction Partners of Calpain-5 Suggest Clues to Its Involvement in Neovascular Inflammatory Vitreoretinopathy
Source: Cells. 2026 Jan 13;15(2):142. doi: 10.3390/cells15020142 (PMC12839053; doi:10.3390/cells15020142)
Supplement: Supplementary file 1 [file cells-15-00142-s001.zip › Supplementary figure legends.pdf]

Supplementary Figure legends for manuscript "Candidate Interaction Partners of Calpain-5 Suggest Clues to Its Involvement in Neovascular Inflammatory Vitreoretinopathy" by Jozsef Gal et al.

Uniform legend for Figures S1-S17: In vitro CAPN5 assays of selected CAPN5 interaction partners identified in this study (Tables 7-8). SH-SY5Y cells were transfected with the indicated expression constructs or their respective vector controls. The cellular lysates were subjected to anti-FLAG immunoprecipitation, and the bound proteins were eluted among native conditions with 3×FLAG peptide. Aliquots of the eluates were incubated with or without activating CAPN5 by the addition of $CaCl_2$ followed by denaturing protein gel electrophoresis and immunoblotting with the indicated antibodies. Prominent proteolytic fragments are denoted by asterisks (where applies). The bars indicate molecular weight marker bands (kDa).

CAPN5_SWATH_uncropped_images.zip: The uncropped immunoblotting images used to prepare Figure 2 and Figures S1-S17 as a compressed folder. Each image has explanatory notes embedded in the image.
