# Supplementary figures and images for: Candidate Interaction Partners of Calpain-5 Suggest Clues to Its Involvement in Neovascular Inflammatory Vitreoretinopathy
Source: Cells. 2026 Jan 13;15(2):142. doi: 10.3390/cells15020142 (PMC12839053; doi:10.3390/cells15020142)

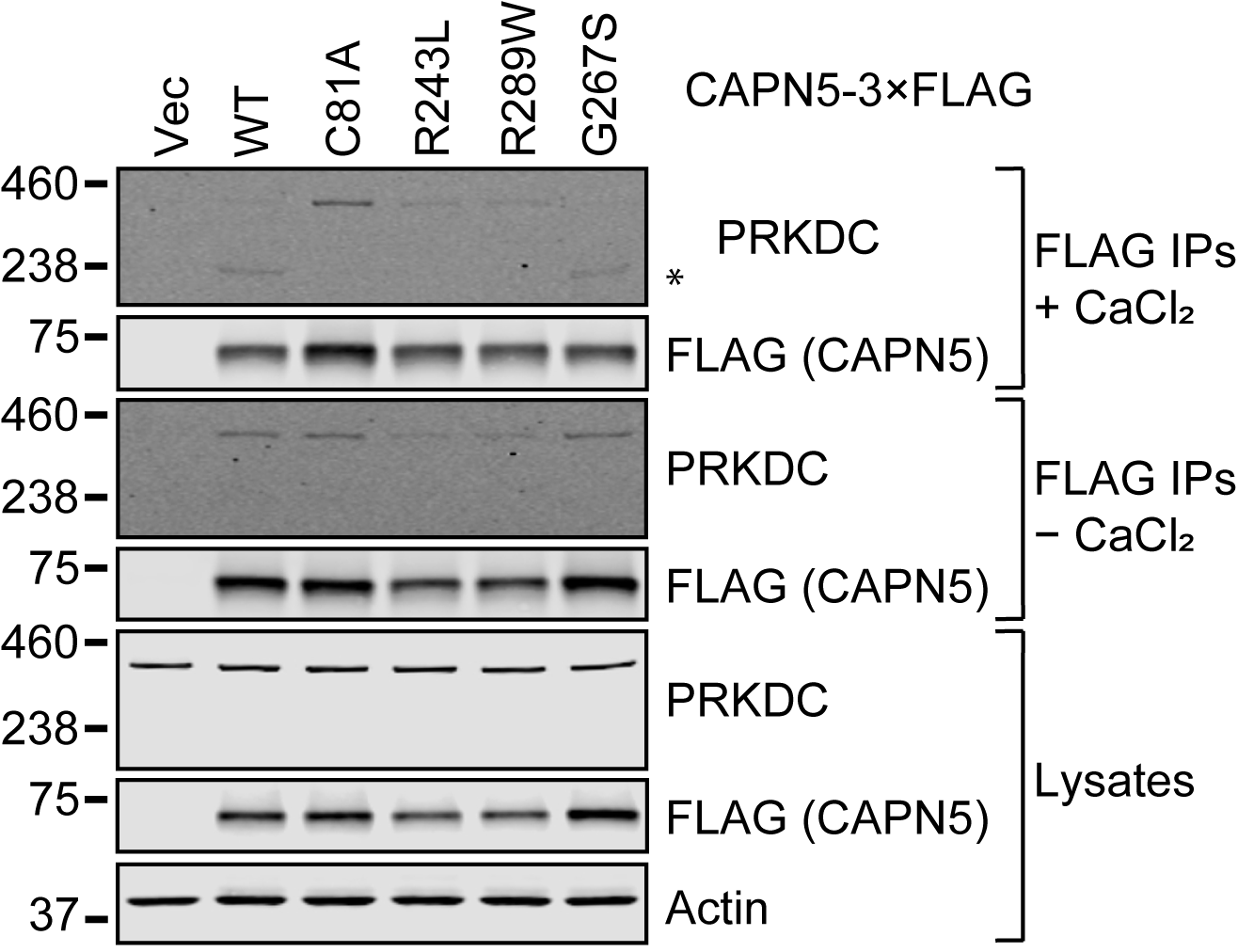

Supplement: Supplementary file 1 [file cells-15-00142-s001.zip › Figures S1-S17/Figure S1 PRKDC.tif]

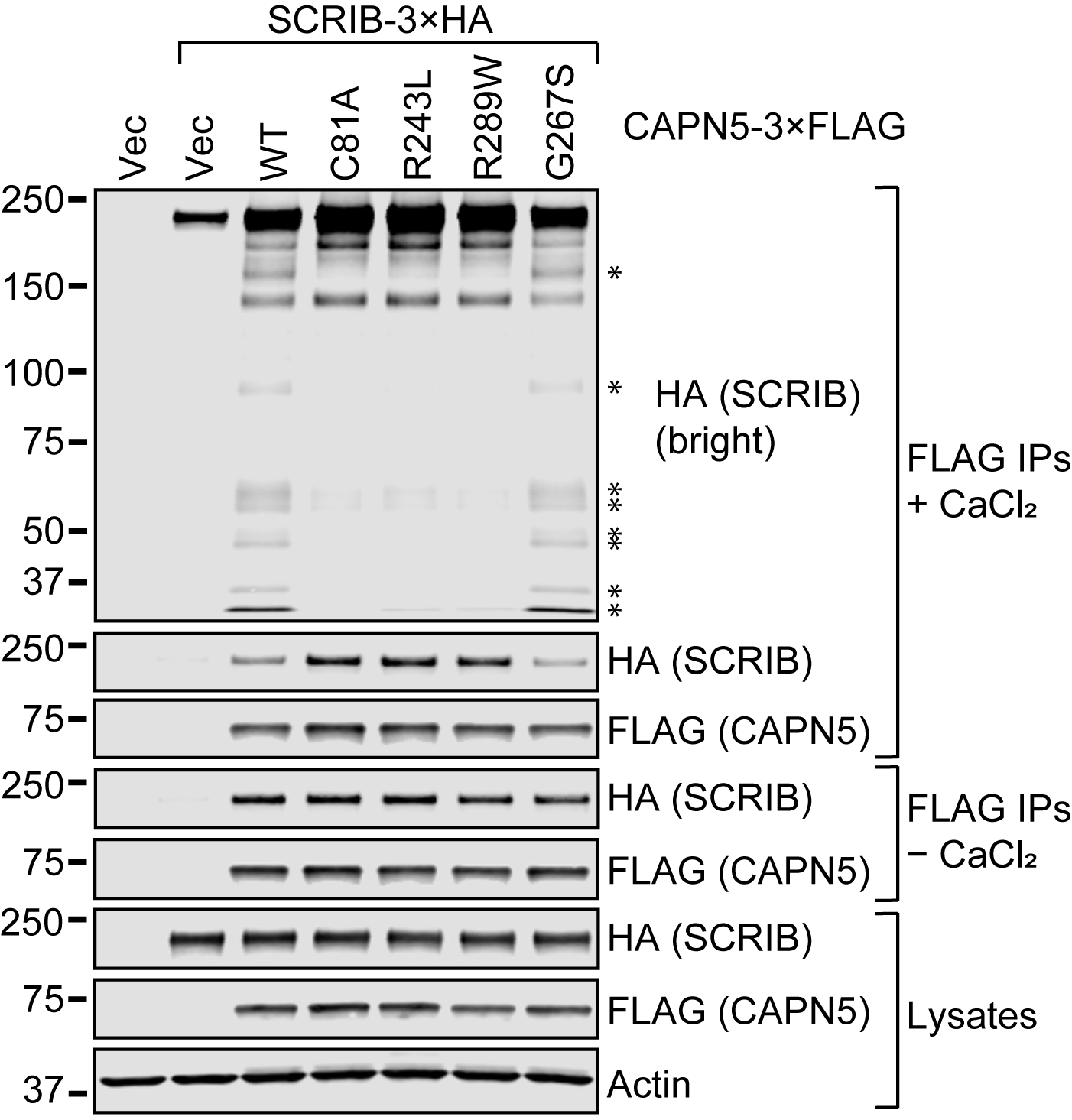

Supplement: Supplementary file 1 [file cells-15-00142-s001.zip › Figures S1-S17/Figure S10 SCRIB-3xHA.tif]

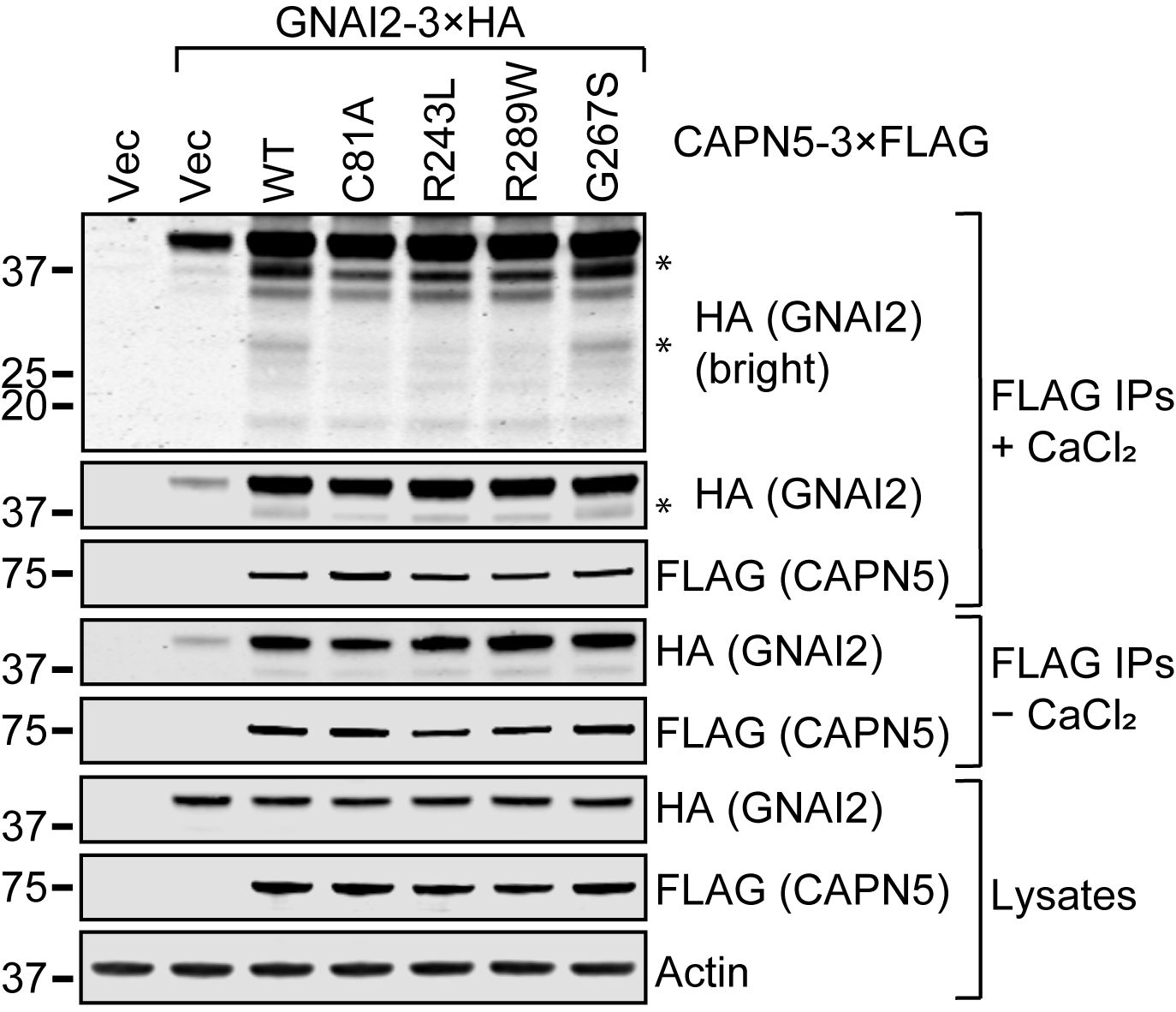

Supplement: Supplementary file 1 [file cells-15-00142-s001.zip › Figures S1-S17/Figure S11 GNAI2-3xHA.tif]

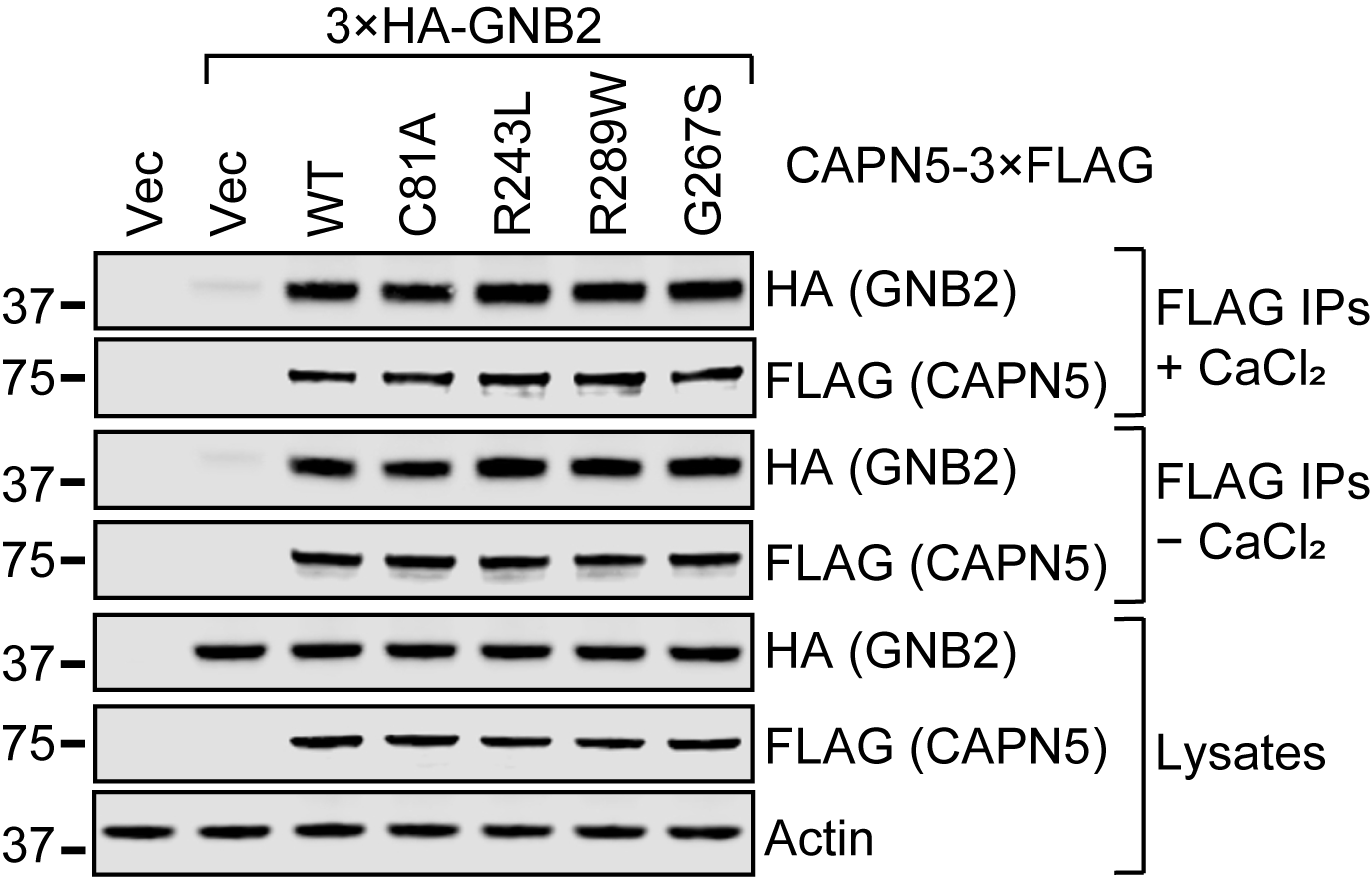

Supplement: Supplementary file 1 [file cells-15-00142-s001.zip › Figures S1-S17/Figure S12 3xHA-GNB2.tif]

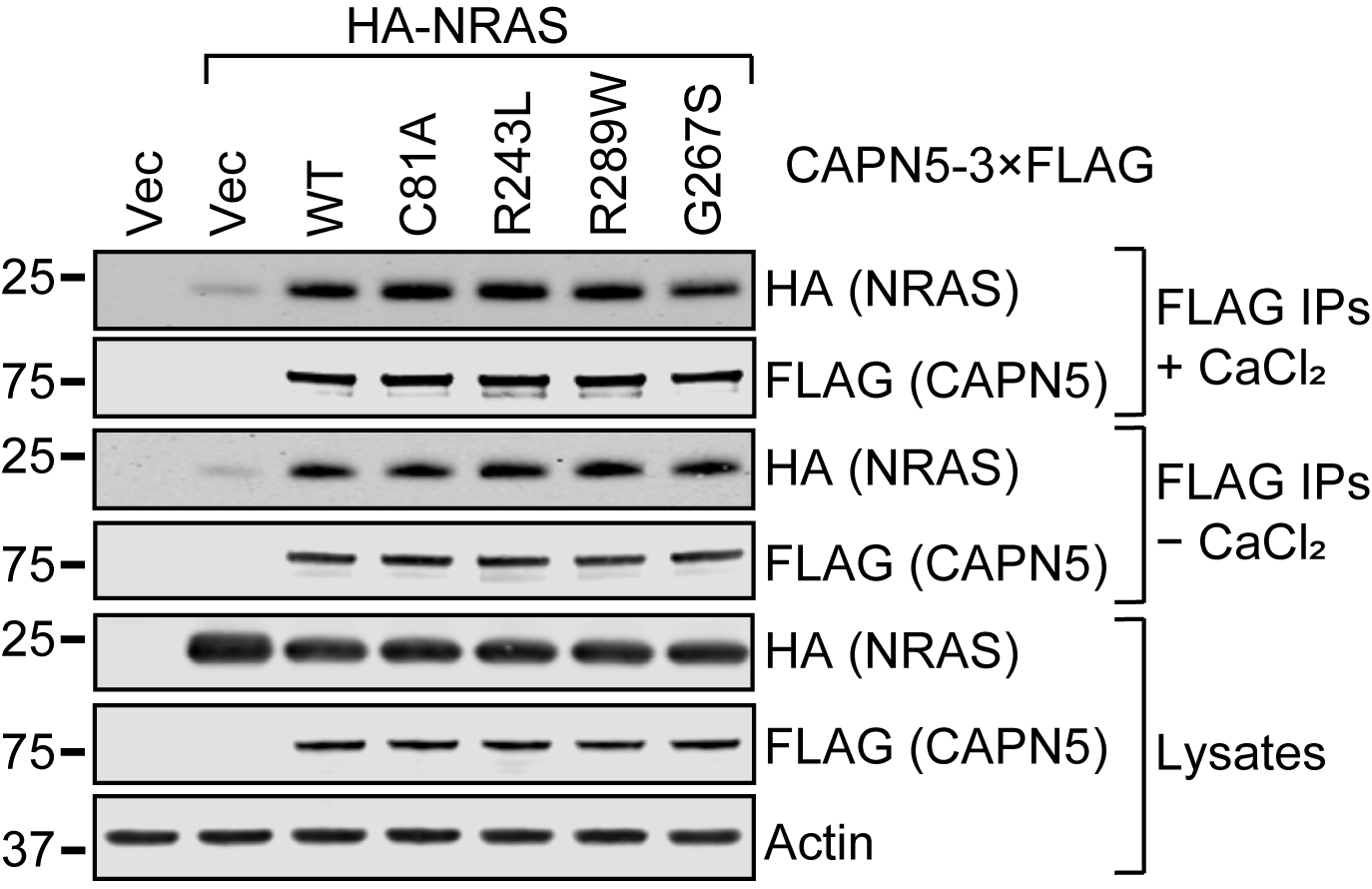

Supplement: Supplementary file 1 [file cells-15-00142-s001.zip › Figures S1-S17/Figure S13 HA-NRAS.tif]

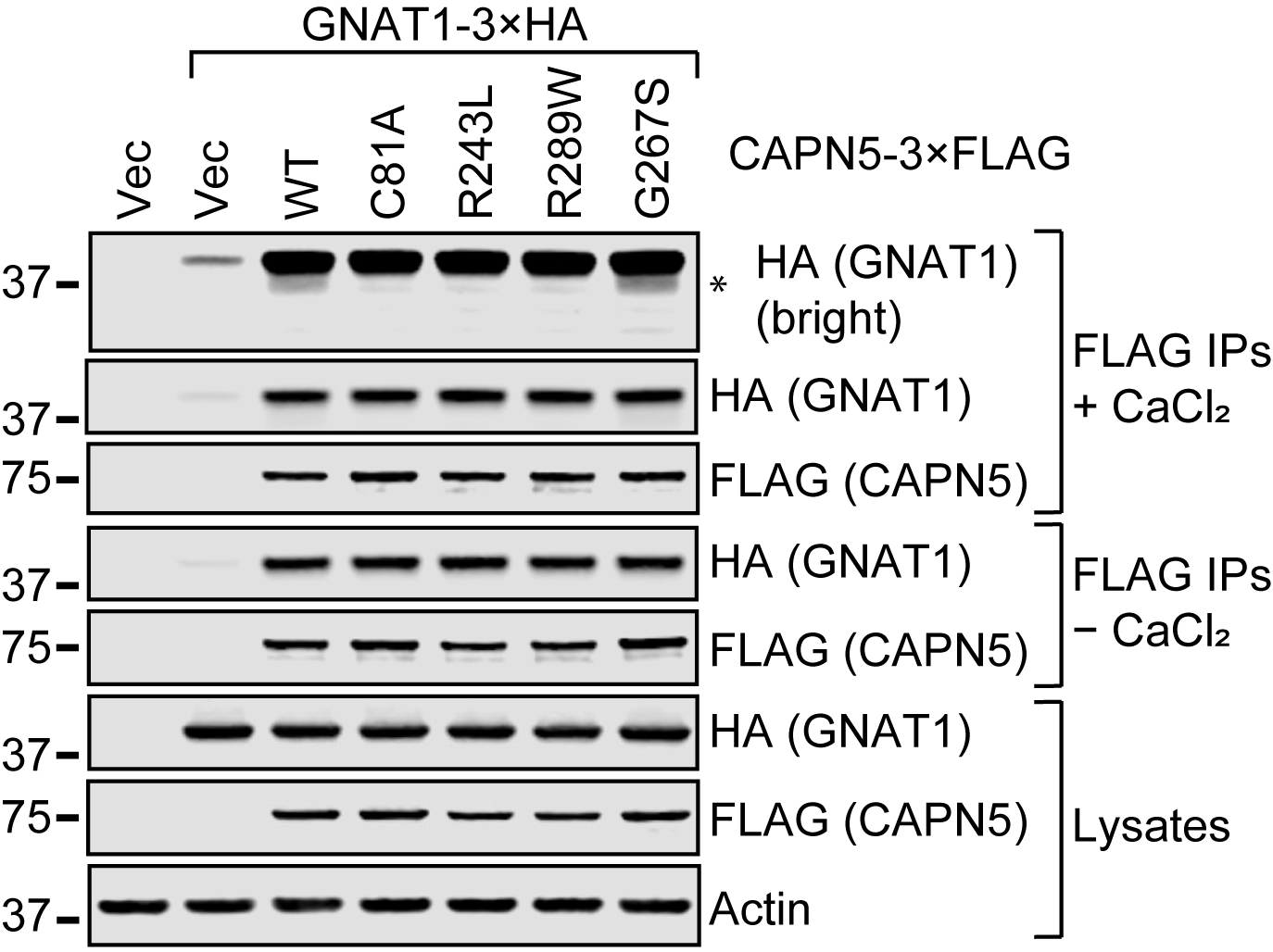

Supplement: Supplementary file 1 [file cells-15-00142-s001.zip › Figures S1-S17/Figure S14 GNAT1-3xHA.tif]

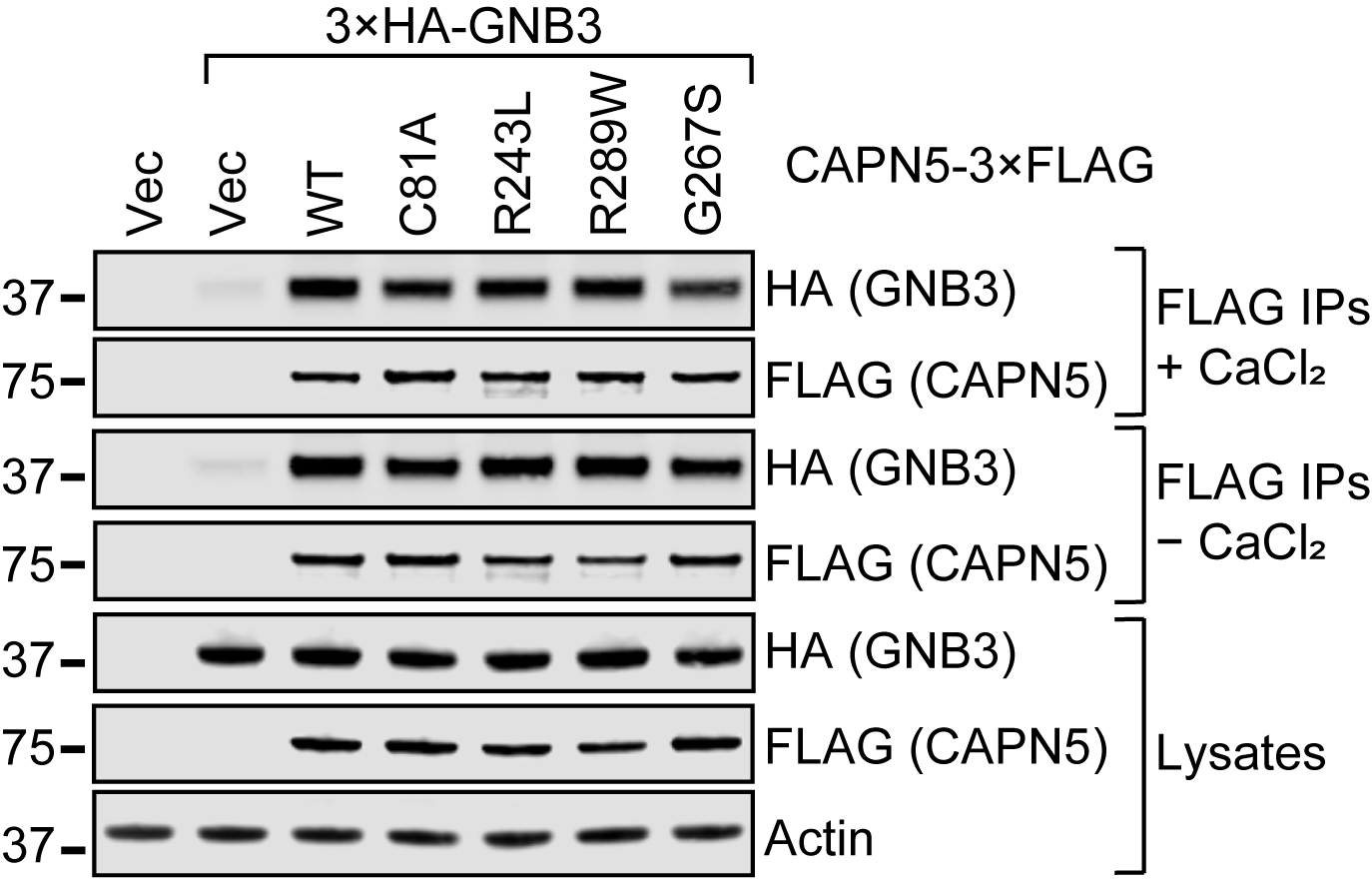

Supplement: Supplementary file 1 [file cells-15-00142-s001.zip › Figures S1-S17/Figure S15 3xHA-GNB3.tif]

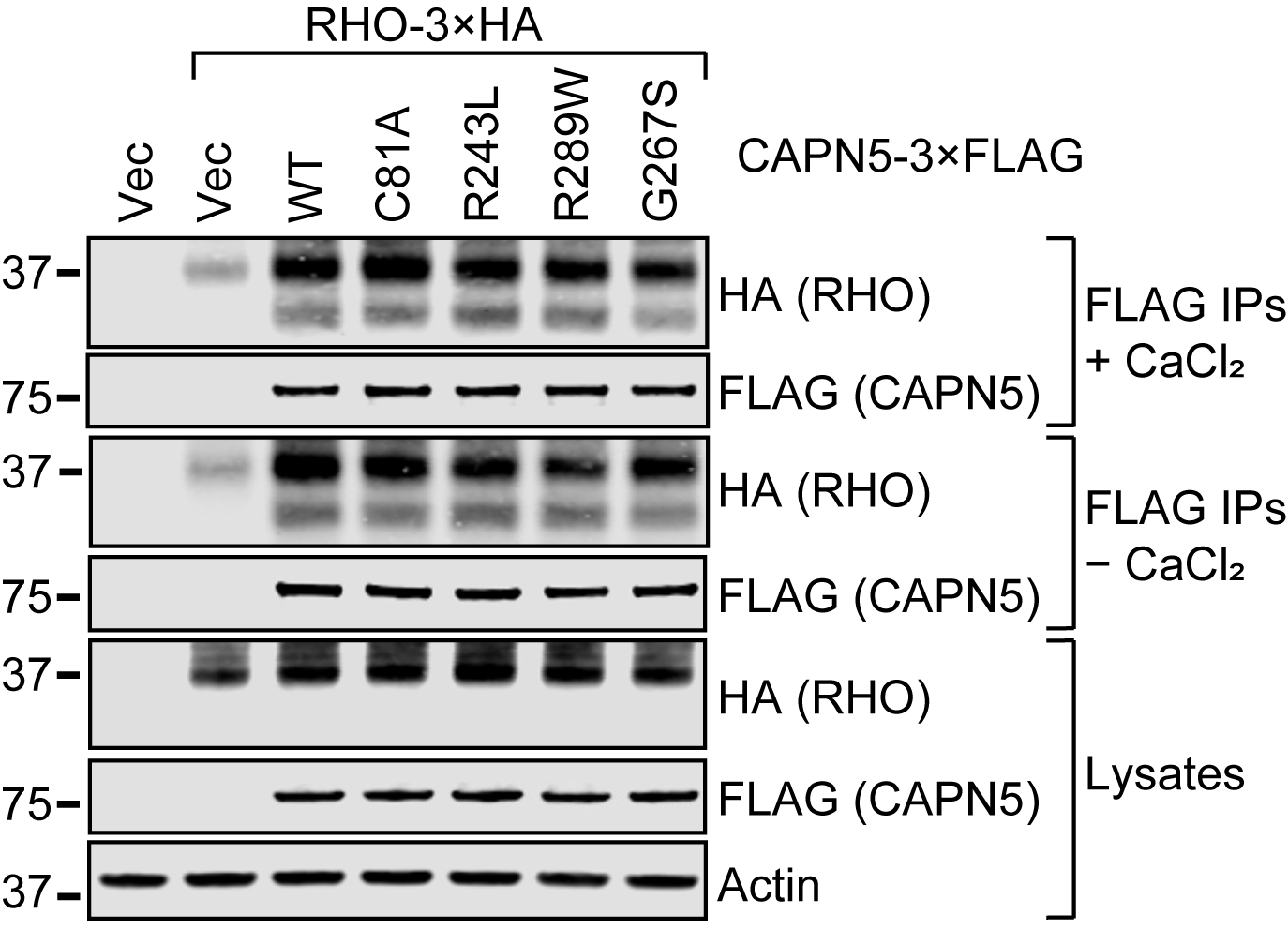

Supplement: Supplementary file 1 [file cells-15-00142-s001.zip › Figures S1-S17/Figure S16 RHO-3xHA.tif]

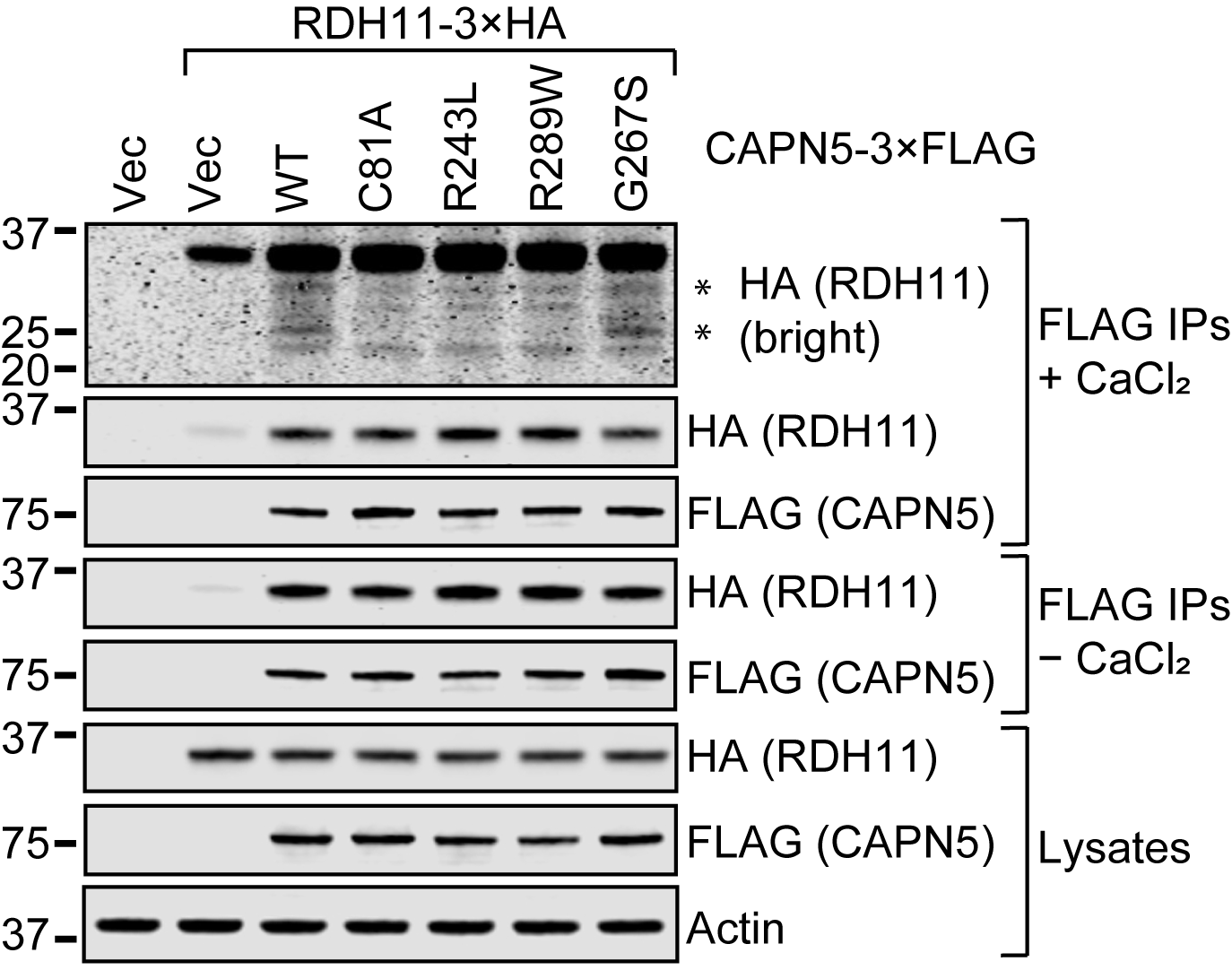

Supplement: Supplementary file 1 [file cells-15-00142-s001.zip › Figures S1-S17/Figure S17 RDH11-3xHA.tif]

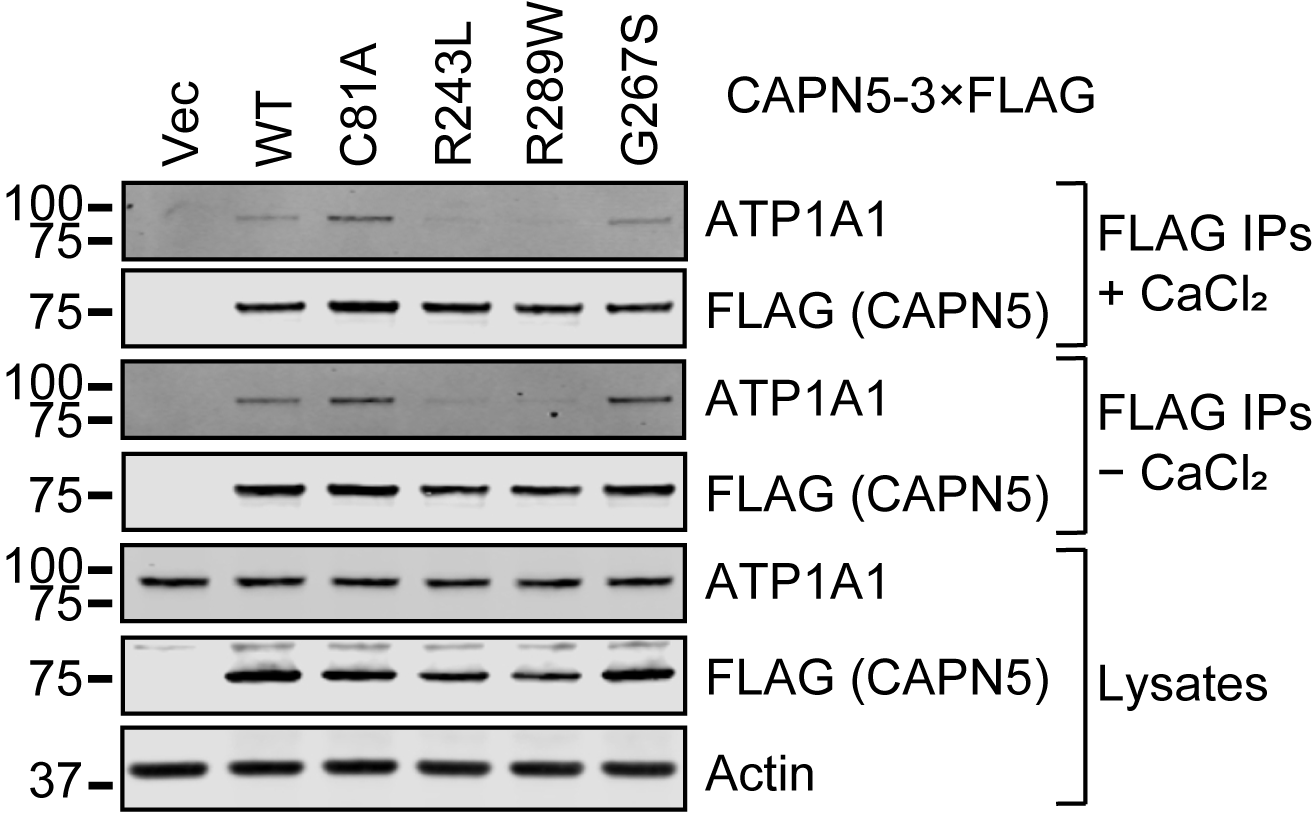

Supplement: Supplementary file 1 [file cells-15-00142-s001.zip › Figures S1-S17/Figure S2 ATP1A1.tif]

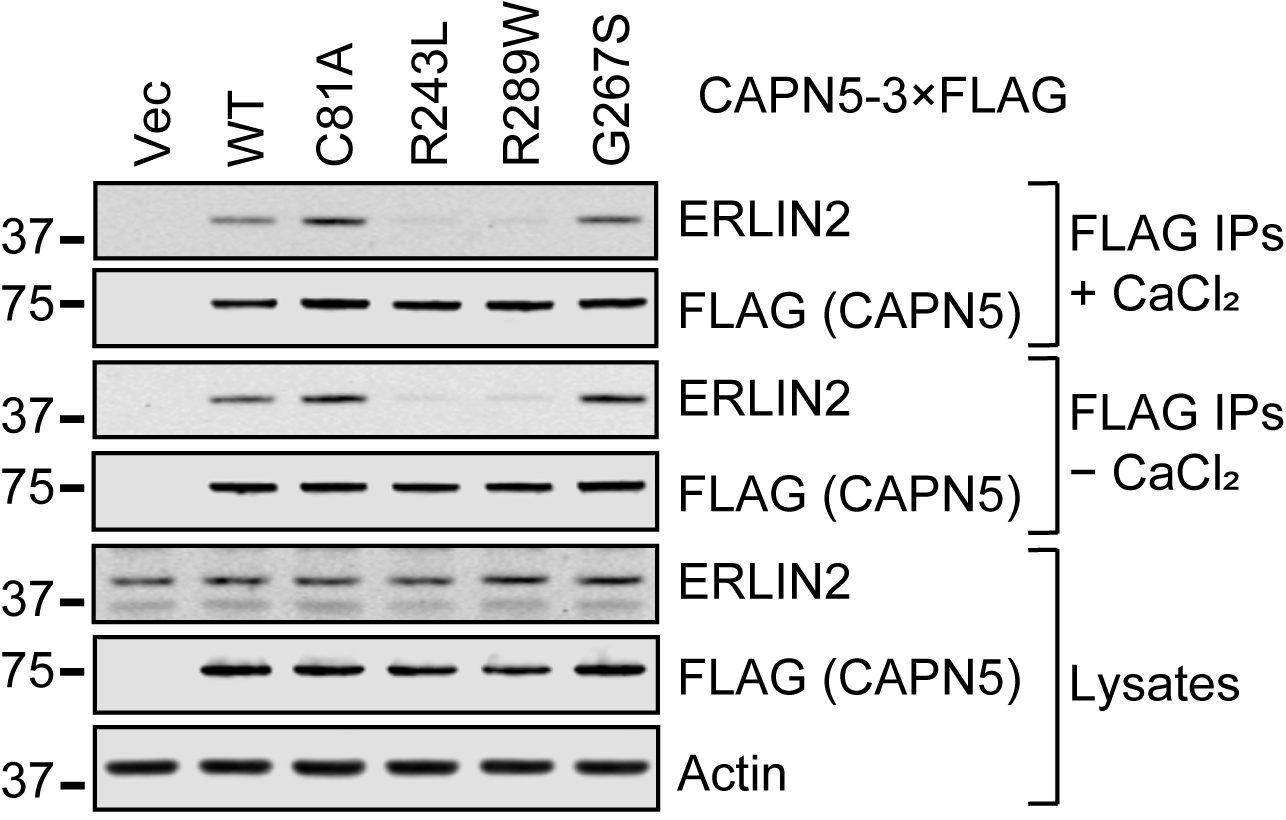

Supplement: Supplementary file 1 [file cells-15-00142-s001.zip › Figures S1-S17/Figure S3 ERLIN2.tif]

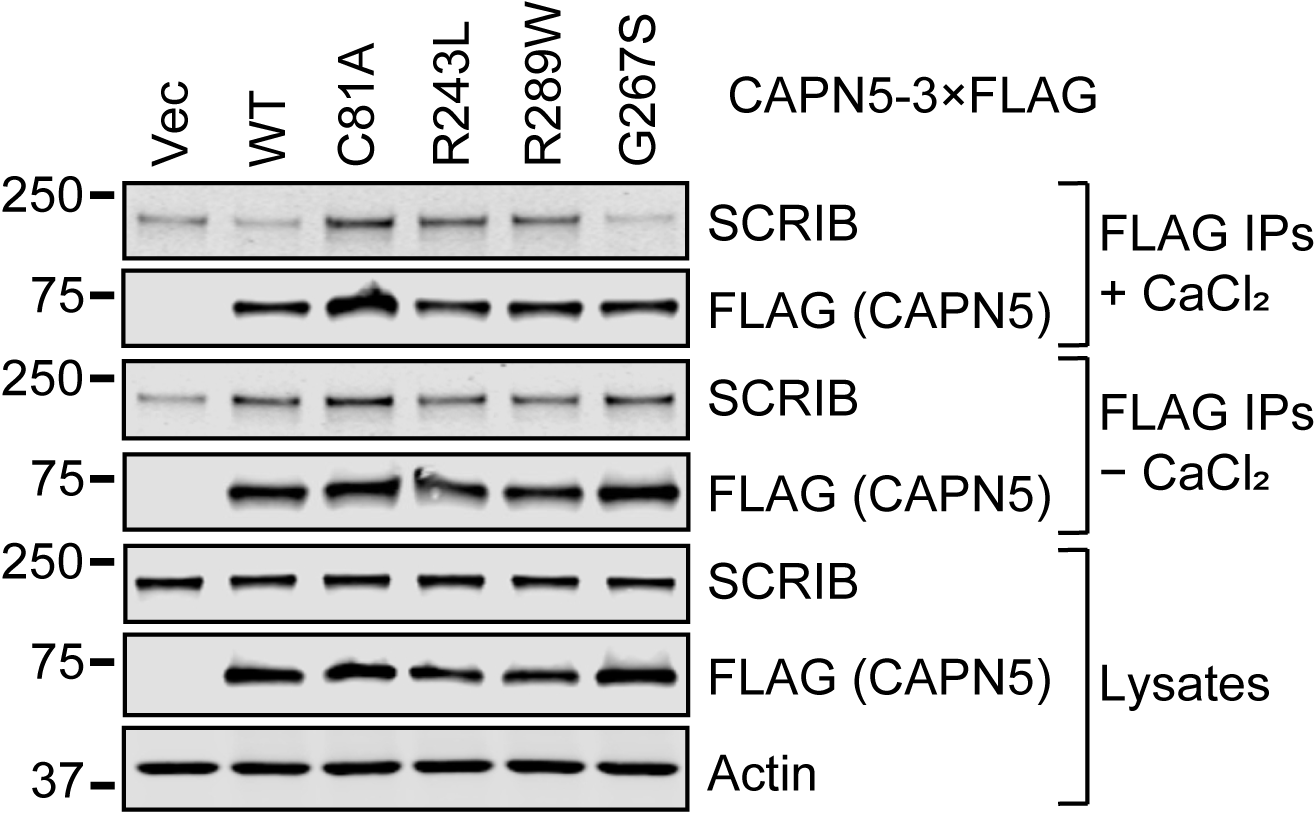

Supplement: Supplementary file 1 [file cells-15-00142-s001.zip › Figures S1-S17/Figure S4 SCRIB.tif]

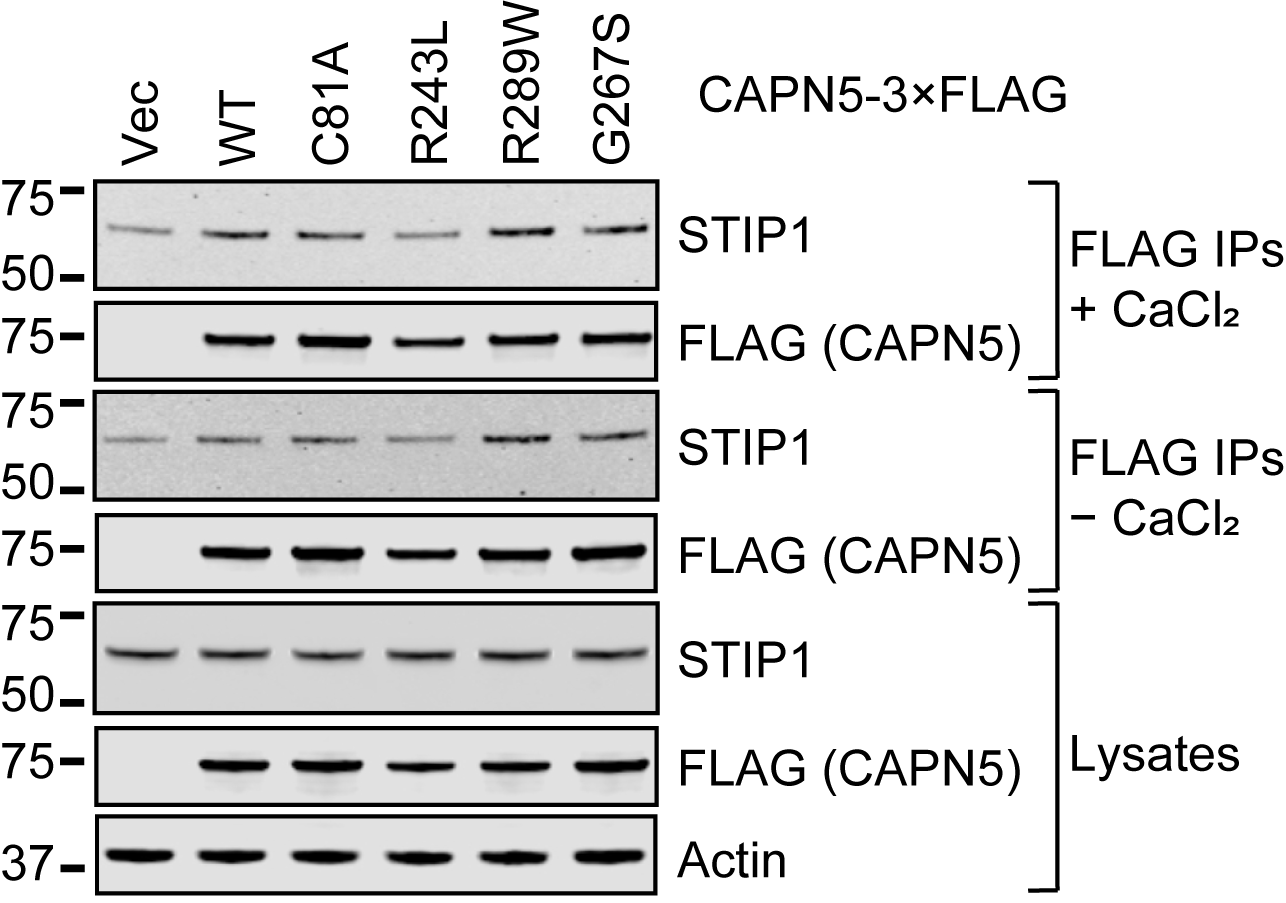

Supplement: Supplementary file 1 [file cells-15-00142-s001.zip › Figures S1-S17/Figure S5 STIP1.tif]

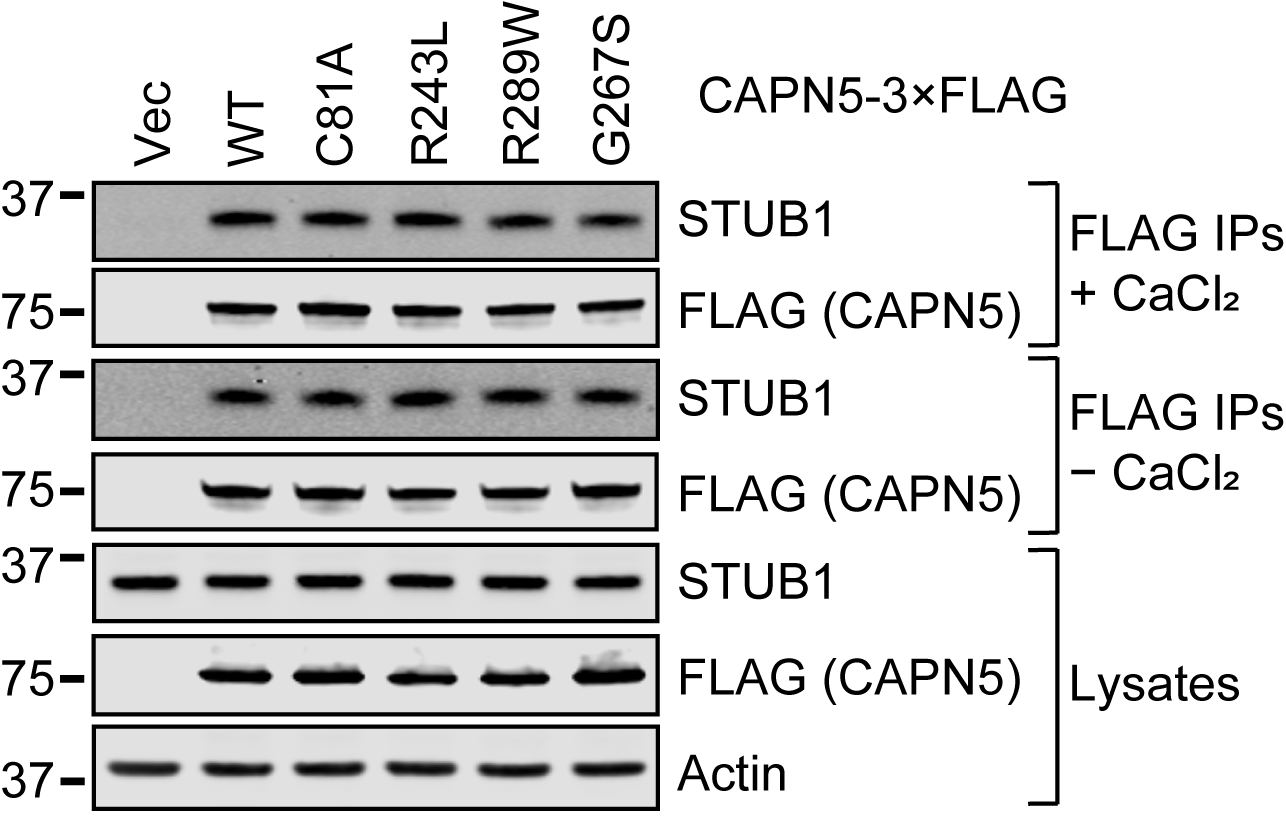

Supplement: Supplementary file 1 [file cells-15-00142-s001.zip › Figures S1-S17/Figure S6 STUB1.tif]

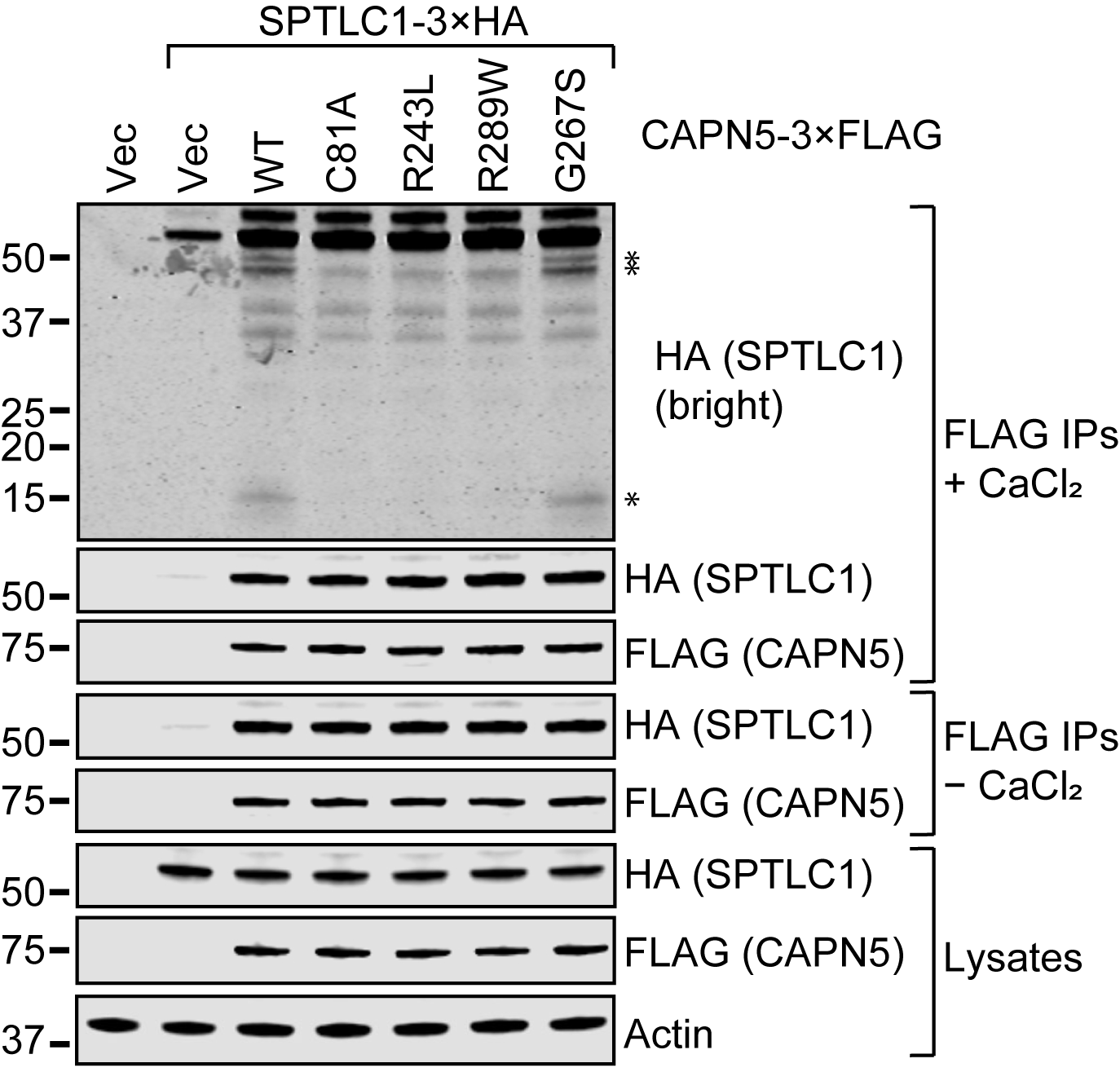

Supplement: Supplementary file 1 [file cells-15-00142-s001.zip › Figures S1-S17/Figure S7 SPTLC1-3xHA.tif]

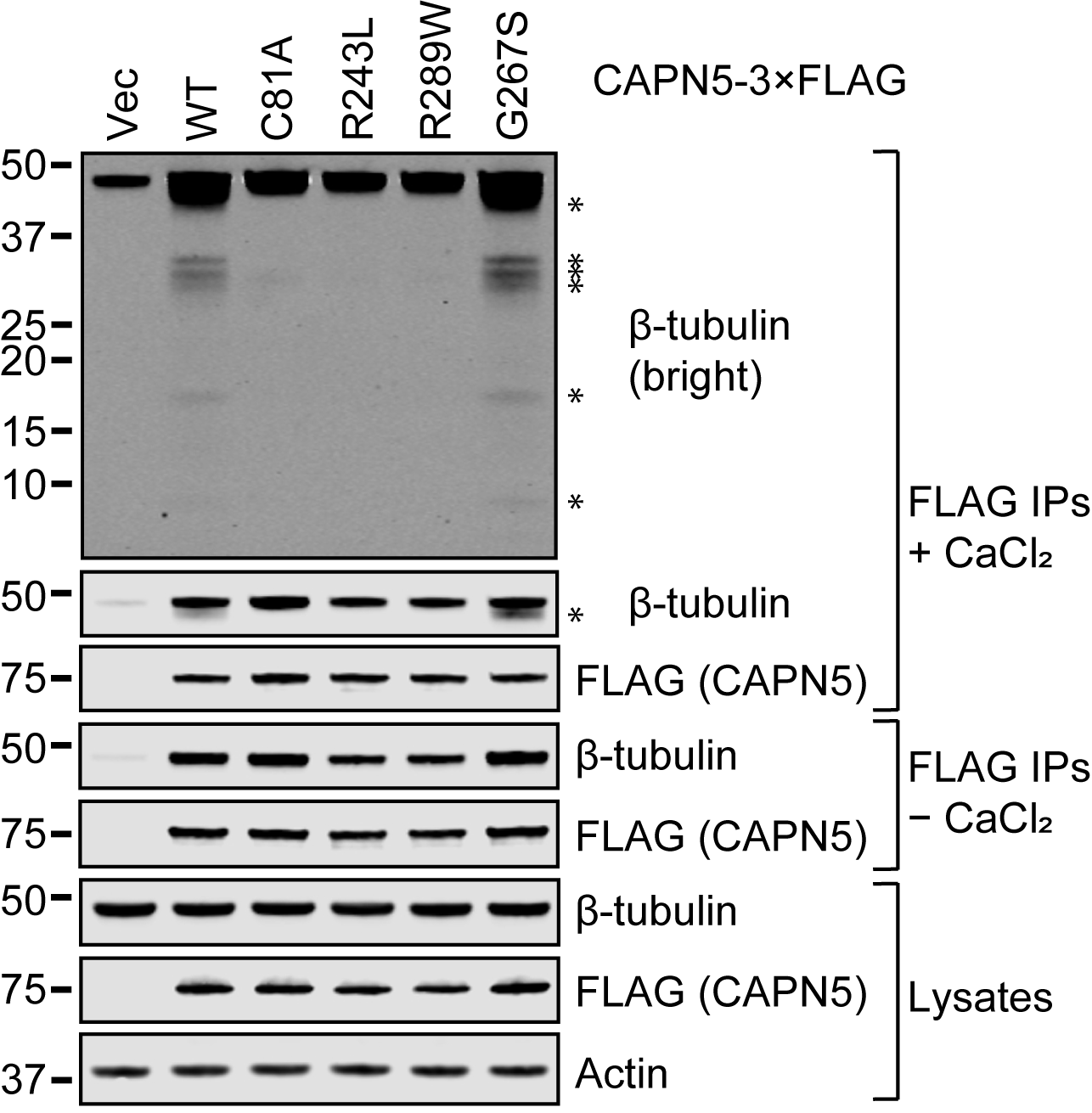

Supplement: Supplementary file 1 [file cells-15-00142-s001.zip › Figures S1-S17/Figure S8 Beta-Tubulin.tif]

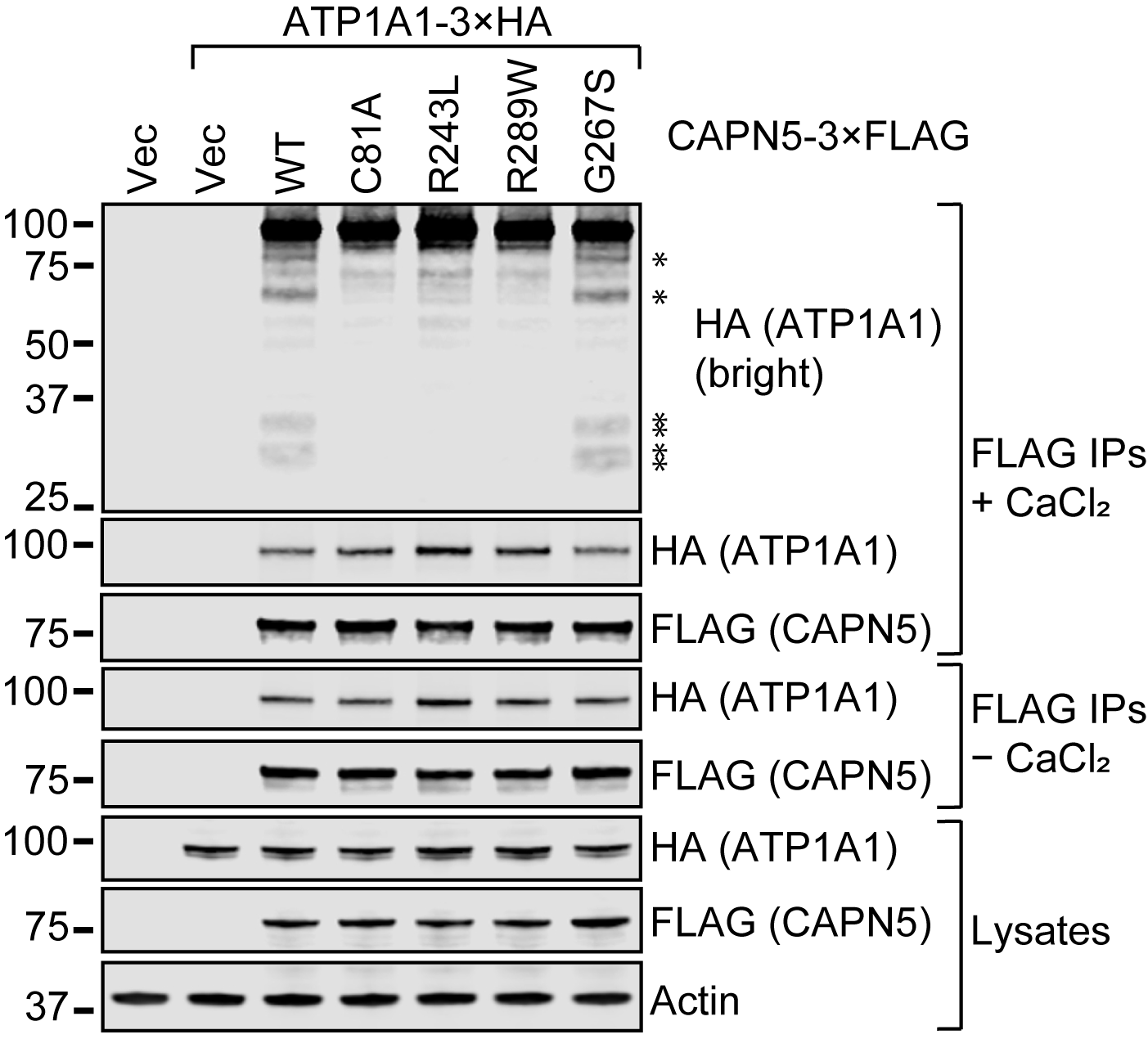

Supplement: Supplementary file 1 [file cells-15-00142-s001.zip › Figures S1-S17/Figure S9 ATP1A1-3xHA.tif]
